# Supplementary material for: Multi-species annotation of transcriptome and chromatin structure in domesticated animals
Source: BMC Biol. 2019 Dec 30;17:108. doi: 10.1186/s12915-019-0726-5 (PMC6936065; doi:10.1186/s12915-019-0726-5)
Supplement: Supplementary file 1 — Additional file 1 Additional file 1: Supplementary figures (S1-S26) and tables (S1-S15). [file 12915_2019_726_MOESM1_ESM.pdf]

Transcriptome and chromatin structure annotation of liver, CD4+ and CD8+ T cells from four livestock species

Supplementary file 1: Supplementary figures and tables

List of Figures

S1 **RNA-seq processing pipeline overview.** RNA-seq reads were processed with STAR for mapping and Cufflinks for transcript modeling using the reference gene annotation as input and for each sample individually. Modelled transcripts were then merged with Cuffmerge to produce a single gene annotation per species, and the transcripts from this novel gene set were further processed with FEELnc to produce a reliable set of lncRNAs. Reference and novel transcripts and gene expressions were quantified in each sample using the STAR/RSEM pipeline. Differential expression analysis was performed on reference and novel genes with TPM $\geq$ 0.1 in at least 2 samples using the R/Bioconductor package **edgeR**. 5

S2 **RNA-seq mapped read classification into genomic domains.** For each sample, RNA-seq mapped reads were classified into exonic (1_exon), intronic (2_intr), intergenic (3_ig) and other (4_other) reads. 6

S3 **RNA-seq sample PCA based on the expression of reference genes.** Only reference genes with a TPM $\geq$ 0.1 in at least 2 samples were used. 7

S4 **Gene expression ratio in males versus females on chromosomes X or Z.** Gene expression was compared in males vs. females using the base 10 logarithm of the TPM values. y-axis: the $\log_2$ of the male/female expression ratio for the genes located on the chromosome of interest. For each plot, the median of these expression ratios is indicated in the title. Left: genes located on chromosome 1 used as a control. Right: genes located on chromosome X for mammals and Z for chicken. x-axis: the position of the gene on the chromosome of interest. 8

S5 **Biological Process GO term enrichment analysis for reference genes differentially expressed between liver and T cells.** This analysis was performed for each species individually (in columns: cattle, goat, chicken, pig in that order) and for genes over-expressed in liver (top) and over-expressed in T cells (bottom). 9

S6 **Molecular Function GO term enrichment analysis for reference genes differentially expressed between liver and T cells.** This analysis was performed for each species individually (in columns: cattle, goat, chicken, pig in that order) and for genes over-expressed in liver (top) and over-expressed in T cells (bottom). 10

S7 **Cellular Compartment GO term enrichment analysis for reference genes differentially expressed between liver and T cells.** This analysis was performed for each species individually (in columns: cattle, goat, chicken, pig in that order) and for genes over-expressed in liver (top) and over-expressed in T cells (bottom). 11

S8 **GO term enrichment analysis for reference genes differentially expressed between liver and T cells in all species.** This analysis was performed for each GO (in column: Biological Process, Molecular Function, Cellular Compartment in that order) and for genes over-expressed in liver (top) and over-expressed in T cells (bottom). 12

S9 **Distribution of FR-AgENCODE transcripts into four positional (known, extension, alternative, novel) and three coding (mRNA, lncRNA, otherRNA) classes. (A)** Distribution of all FR-AgENCODE transcripts into positional and coding classes; **(B)** and **(C)** Distribution of FR-AgENCODE transcripts of each coding class into positional classes, and of each positional class into coding classes respectively. 13

S10 **Novel coding FR-AgENCODE genes enrich the set of blood and T cell annotated genes.** (A) Venn diagrams of novel coding genes for each triplet of livestock species (two livestock genes are defined as orthologous if they project to the same human gene). (B) Gene set enrichment analysis on the human orthologs of the 93 cattle, 52 goat, 74 chicken and 26 pig genes, using EnrichR (Kuleshov et al. 2016; `http://amp.pharm.mssm.edu/Enrichr/`) on the ARCS4 Tissue dataset (`https://amp.pharm.mssm.edu/archs4/`). (C) Gene names of the 12 genes that are common to two livestock species. Out of these 12, 8 are coding for T cell Receptor Alpha or Beta Variable genes. 14

S11 **RNA-seq sample PCA based on FR-AgENCODE gene expression.** 15

S12 **LncRNA gene features.** Genomic structure features (left) and expression (right) are indicated for protein coding genes (coding) and lncRNA genes (lnc). The expression (TPM) is given for the three tissues and the four species; lncRNA genes are between 8- and 43-fold less expressed than protein coding genes (expression median: 0.4 vs. 10 TPM respectively). . . . 16

S13 **Syntenic lncRNAs conserved between the 4 livestock species and human.** The 6 syntenic lncRNAs conserved in the 4 livestock species and human are represented in green and their surrounding protein coding genes in orange. Distances between the genes are indicated either in base pair or in kilo base pair (k). A distance of 0 means the genes are overlapping. A distance in red means a lower confidence in the orthology relationship for this species, after inspection of the distances found in the other species. When the lncRNA was not known before, a new gene name is proposed (asterisk). 17
S14 **ATAC-seq read pair summary statistics.** For each species and sample (labelled by its tissue and animal number), the number of initial read pairs (1_init), of read pairs obtained after trimming (2_trim), mapping (3_map), proper pairing (4_pair), q10 filtering (5_q10), mitochondrial read removal (6_nonmt), and PCR duplicate read removal (7_nondup) are shown. 18
S15 **ATAC-seq peak size distribution.** For each species, the size distribution of tissue and merged (i.e. across all tissues) ATAC-seq peaks are provided. 19
S16 **Density of ATAC-seq peaks around starts of novel (i.e. not known) transcripts for cattle (A), goat (B), chicken (C) and pig (D)** 20
S17 **ATAC-seq peak distribution into genomic domains.** For each species, are provided: the percentage of ATAC-seq peaks that are exonic, intronic, overlapping the TSS of a reference gene extended by 0, 1 or 5 Kb on each side, overlapping the TTS of a reference gene extended by 0, 1 or 5 Kb on each side and intergenic. 21
S18 **Per sample promoter accessibility for four reference gene expression quartiles in pig.** 22
S19 **Schematic illustration of the per-gene and cross-sample RNA-seq versus ATAC-seq correlation analysis.** 23
S20 **Correlation between reference gene expression and chromatin accessibility at the TSS for goat.** For each pair of reference gene and ATAC-seq peak overlapping the reference gene TSS extended by 1 Kb on each side, the Pearson correlation was computed between the base 10 logarithm of the gene TMM and the base 10 logarithm of the normalized ATAC-seq signal at the peak. The distribution was then plotted for non DE genes **(A, top)** and for DE genes **(B, bottom)**. 24
S21 **ATAC-seq sample heatmap and hierarchical clustering based on the 1083 level 4 ATAC-seq peaks.** Pairwise similarity between samples is computed as the Pearson correlation between the base 10 logarithm of the normalized reads of the 1083 ATAC-seq peaks common to 4 species. These similarities are plotted as a heatmap, where samples appear both as rows and columns and are labelled by their species, tissue and the sex of the animal. The color of each heatmap cell also reflects the similarity (Pearson correlation) between each sample pair (the lighter, the higher). Hierarchical clustering is performed using one minus the squared Pearson correlation as a distance and the complete linkage aggregation method. 25
S22 **Relation between non promoter proximal chromatin accessibility conservation and differential accessibility.** This figure is the same as main Figure 6 but is done restricting to ATAC-seq peaks not overlapping the TSS+-1kb in any of the species where it is present. Phastcons scores of these ATAC-seq peaks were plotted after dividing the human hits according to both their similarity level (between 1 and 3, x-axis, level 4 removed because there were less than 50 peaks in a category) and their differential accessibility (DA) status (DA in at least one species or DA in none of the 4 species, boxplot color). Although the phastcons score obviously increases with the similarity level, it is clear that, for a given similarity level, the phastcons score is higher for DA human hits than for non DA human hits (all similarity levels, p-values $< 1.2 \times 10^{-06}$ overall, Wilcoxon tests) (number of elements the boxplots from left to right: 151,783, 19,732, 11,922, 3,317, 2,100, 1,082). 26
S23 **Hi-C read summary statistics.** For each species and animal, are plotted the number of: initial read pairs after sequencing (1_initial), pairs with both reads mapped on the genome (2_reported), pairs in a valid configuration, that is when the sum of the distances from the reads to their next HindIII restriction sites downstream is comprised between 20bp and 1Kb (3_valid), pairs after removing PCR duplicates (4_valid_rmdup) and the number of read pairs supporting proximity between two different chromosomes (5_trans). 27

S24 **Method for predicting Hi-C A and B compartments.** Illustration of the A/B compartment calling workflow using the interaction matrices of the first chromosome in pig. Upper panels: interaction matrices at different steps of the workflow. Lower panel: the first three eigenvectors are shown along the chromosome to illustrate the relevance of PC#1 as the discriminative value to segregate bins between A and B compartments. 28
S25 **CTCF motif density and local interaction score (left) and Directionality Index (DI, right) within and around Hi-C Armatus and Armatus/Juicer TADs.** 29
S26 **Distribution of Hi-C A and B compartments along each chromosome and for each animal.** Genome-wide overview of compartment labels per 500Kb bin in pig for each animal. A general coherence can be observed across replicates. White regions are devoid of any called compartment. 30

List of Tables

S1 **Completed experiments.** Available data per animal, sample and experimental assay. Green: completed experiment. Red (NA): not available. Hi-C was attempted on liver samples only, and succeeded on all species but cattle. Consequently, ATAC-seq was not attempted on the cattle liver samples either. 32
S2 **Genome and reference gene annotation used for each species.** 33
S3 **Software used in the FR-AgENCODE project.** 34
S4 **RNA-seq read mapping statistics.** Number and proportion of mapped and uniquely mapped RNA-seq read pairs for each species, replicate and tissue. 36
S5 **Differentially Expressed (DE) reference genes.** Number of differentially expressed reference genes obtained by the two statistical models (see main text and Methods) 37
S6 **Genes consistently over-expressed in CD4+ compared to CD8+ or reciprocally, in four livestock species.** For the 39 genes consistently seen as over-expressed in CD4+ with respect to CD8+ (10 genes) or reciprocally (29 genes), are indicated: the human gene ID, the gene name in a column that indicates the cell type in which the gene is over-expressed, the TPM in human CD4+ and CD8+ cells from the Blueprint project (CD4-positive, alpha-beta T cell and CD8-positive, alpha-beta T cell respectively), whether or not these expression levels are consistent with the differential behavior observed in livestock, the expression in livestock CD4+ and CD8+ (average across samples) and a reference describing the role of the gene in blood cells when available. 38
S7 **FR-AgENCODE transcript positional classification.** 39
S8 **FR-AgENCODE novel coding genes and their orthology with human** 40
S9 **Differentially Expressed (DE) FR-AgENCODE genes.** Number of differentially expressed FR-AgENCODE genes obtained by the two statistical models (see main text and Methods). . 41
S10 **lncRNA classification.** Number of classified expressed lncRNAs per species. This table includes monoexonic lncRNAs that represent 57-68% of these transcripts. Loci are bracketed. Fields with an asterisk (*) indicate the existence of lncRNAs unclassified by FEELnc because they are on unassembled contigs; they represent 217, 717, 2,718 and 83 lncRNAs in cattle, goat, chicken and pig respectively and are not listed here. 42
S11 **FR-AgENCODE transcript coding classification.** 43
S12 **Number of ATAC-seq peaks per species.** 44
S13 **Number of differentially accessible (DA) ATAC-seq peaks per species.** 45
S14 **Hi-C read pair mapping statistics.** Number of read pairs of different categories. *Initial*: total number of sequenced read pairs. *Reported*: pairs with both reads mapped on the genome. *Valid*: uniquely mapped pairs with an estimated insert size (sum of the distances from the reads to their next downstream HindIII genomic sites) between 20bp and 1Kb. *Valid.rmdup*: valid read pairs after duplication removal that were used to build the interaction matrices. *Trans*: pairs with reads on different chromosomes. 46
S15 **Statistics of Hi-C TADs and A/B compartments.** 47

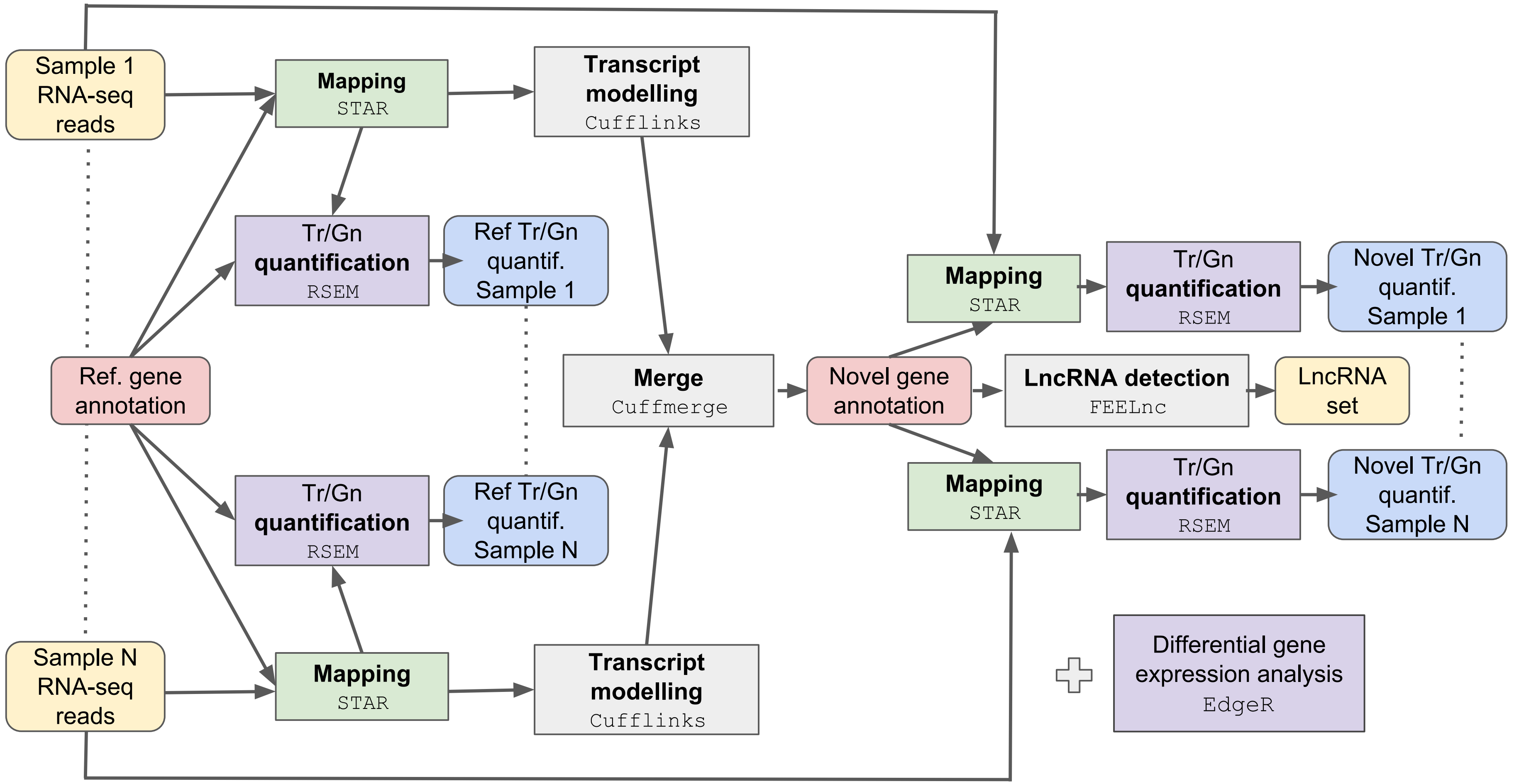

Figure S1: **RNA-seq processing pipeline overview.** RNA-seq reads were processed with STAR for mapping and Cufflinks for transcript modeling using the reference gene annotation as input and for each sample individually. Modelled transcripts were then merged with Cuffmerge to produce a single gene annotation per species, and the transcripts from this novel gene set were further processed with FEELnc to produce a reliable set of lncRNAs. Reference and novel transcripts and gene expressions were quantified in each sample using the STAR/RSEM pipeline. Differential expression analysis was performed on reference and novel genes with TPM $\geq$ 0.1 in at least 2 samples using the R/Bioconductor package **edgeR**.

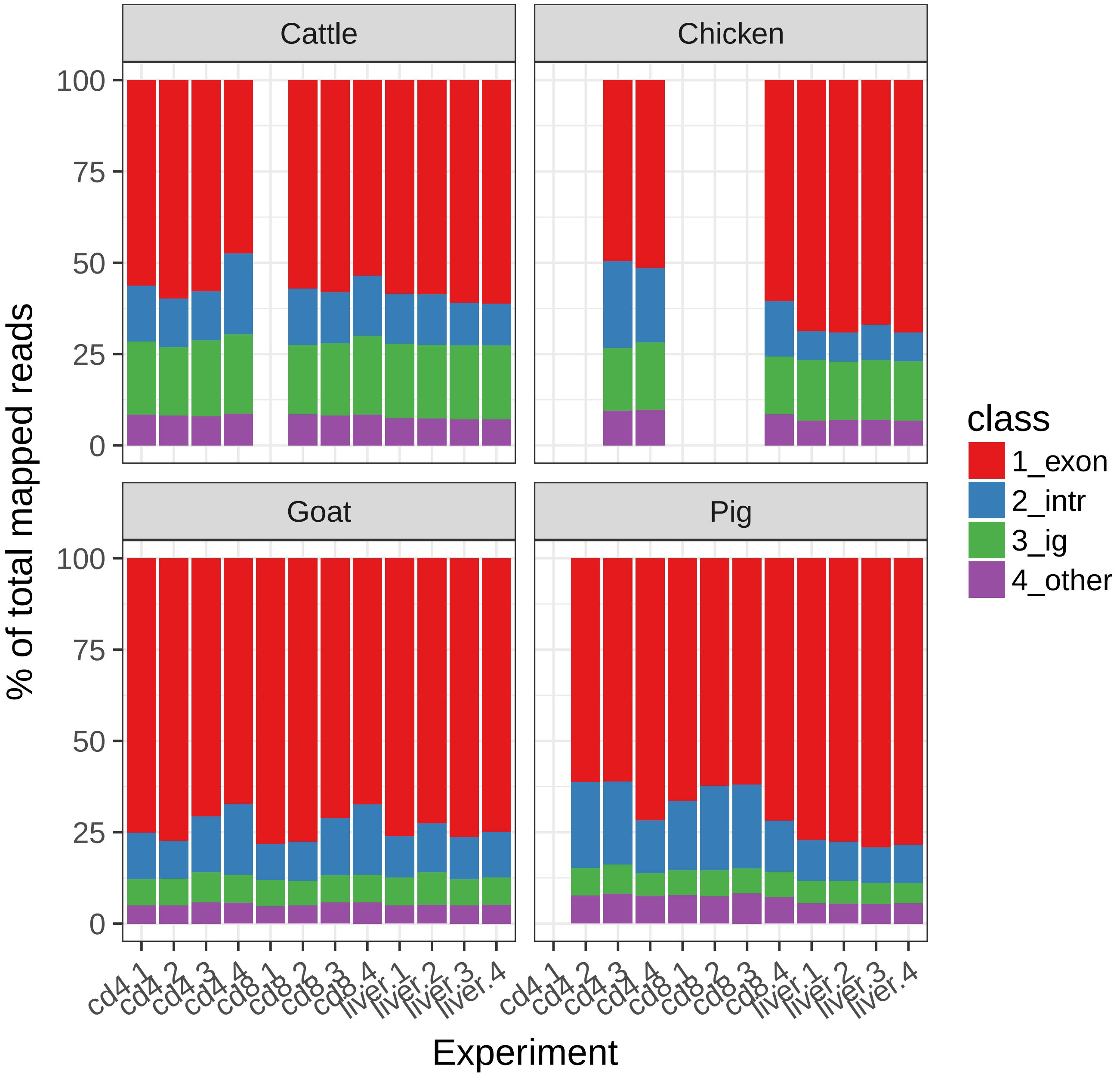

Figure S2: **RNA-seq mapped read classification into genomic domains.** For each sample, RNA-seq mapped reads were classified into exonic (1_exon), intronic (2_intr), intergenic (3_ig) and other (4_other) reads.

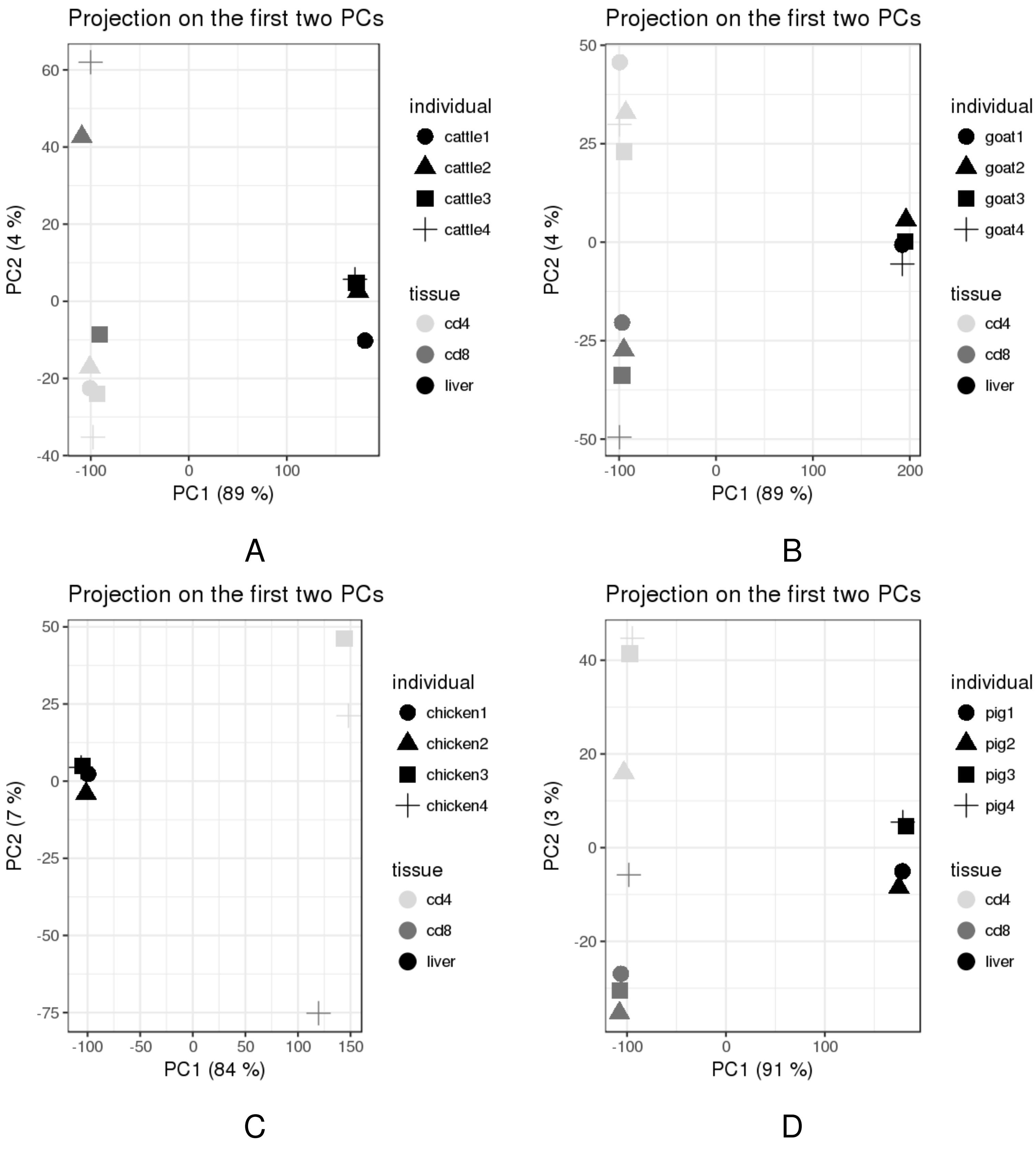

Figure S3: **RNA-seq sample PCA based on the expression of reference genes.** Only reference genes with a TPM $\geq$ 0.1 in at least 2 samples were used.

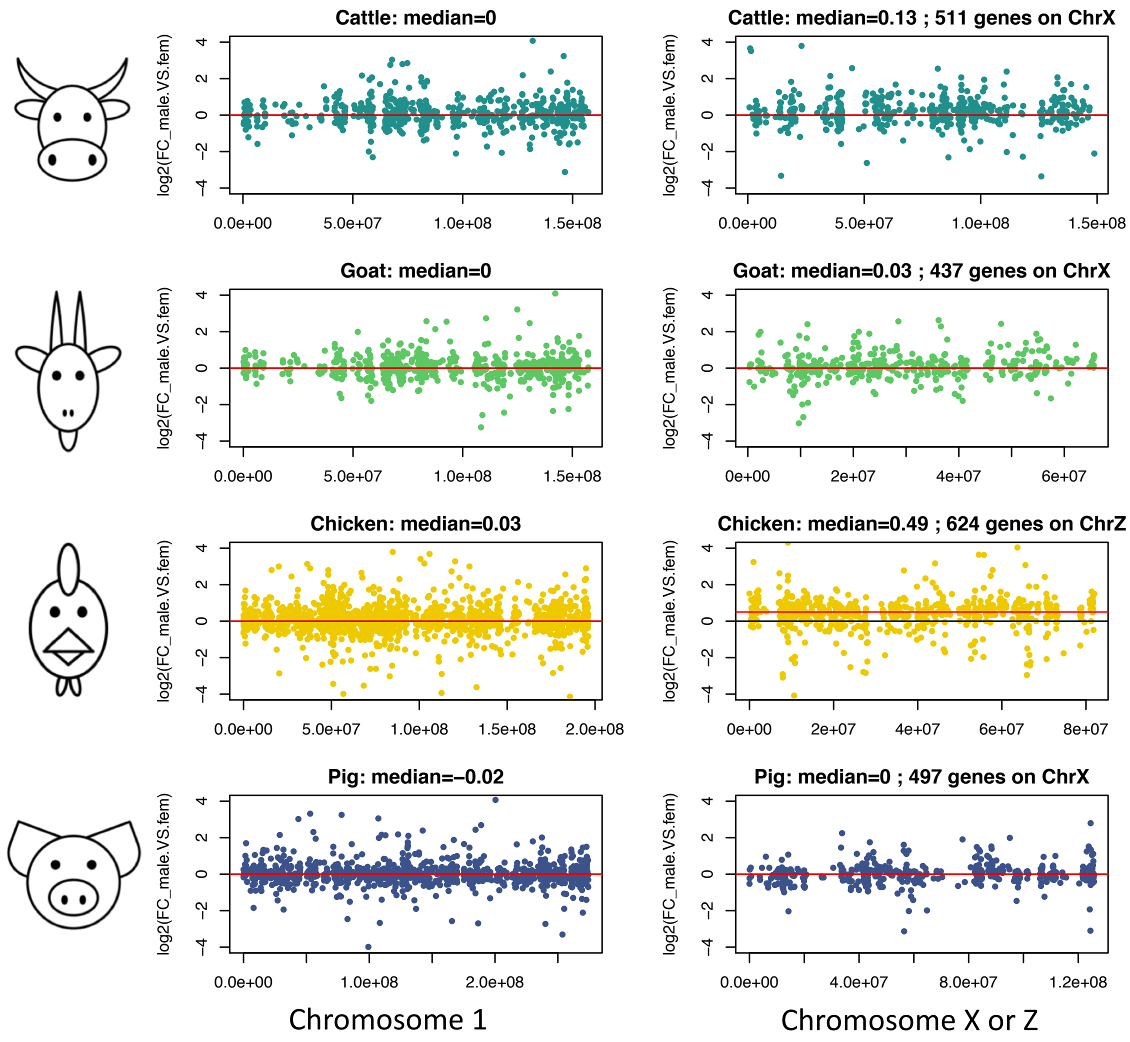

Figure S4: **Gene expression ratio in males versus females on chromosomes X or Z.** Gene expression was compared in males vs. females using the base 10 logarithm of the TPM values. y-axis: the $\log_2$ of the male/female expression ratio for the genes located on the chromosome of interest. For each plot, the median of these expression ratios is indicated in the title. Left: genes located on chromosome 1 used as a control. Right: genes located on chromosome X for mammals and Z for chicken. x-axis: the position of the gene on the chromosome of interest.

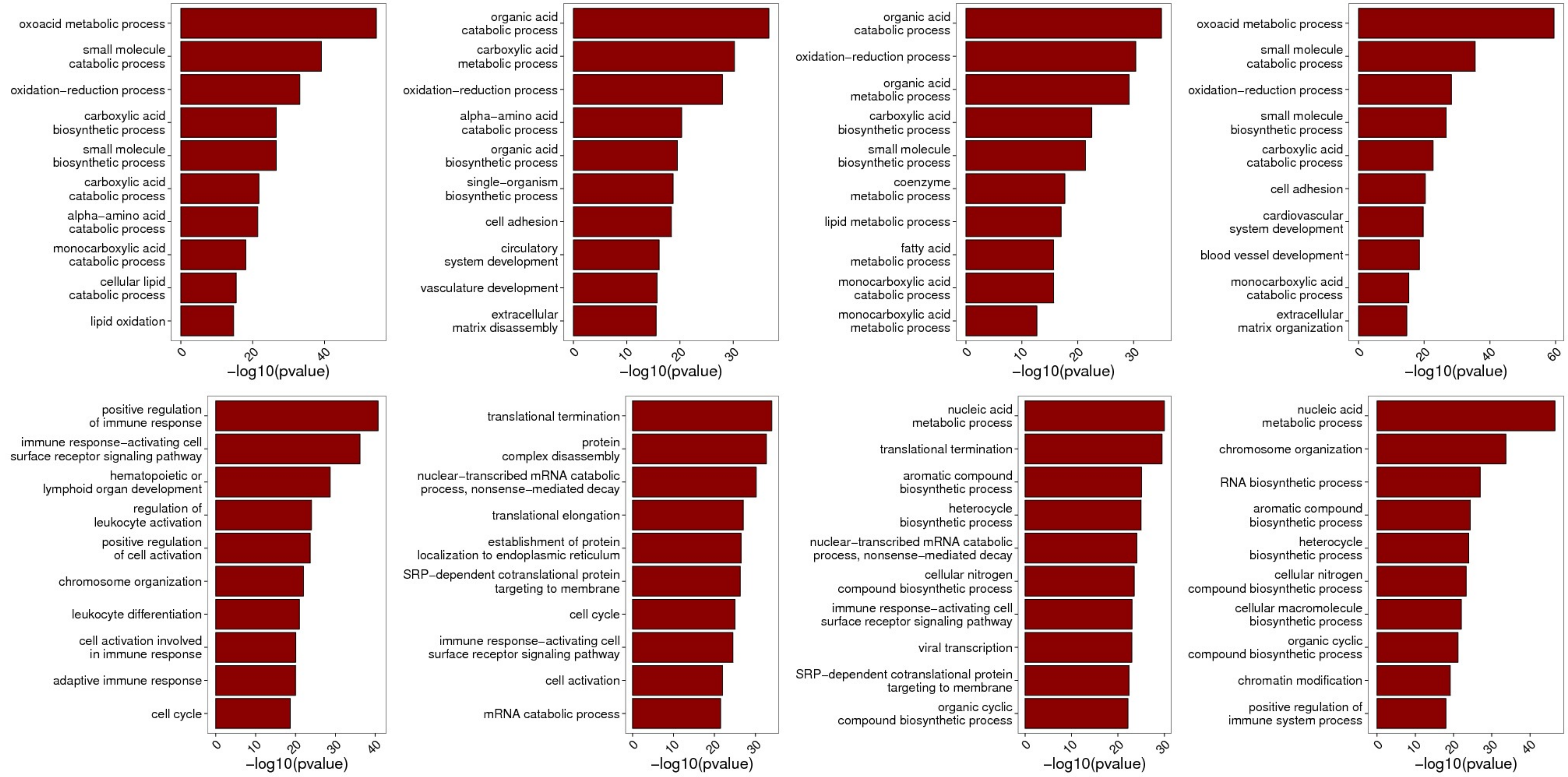

Figure S5: **Biological Process GO term enrichment analysis for reference genes differentially expressed between liver and T cells.** This analysis was performed for each species individually (in columns: cattle, goat, chicken, pig in that order) and for genes over-expressed in liver (top) and over-expressed in T cells (bottom).

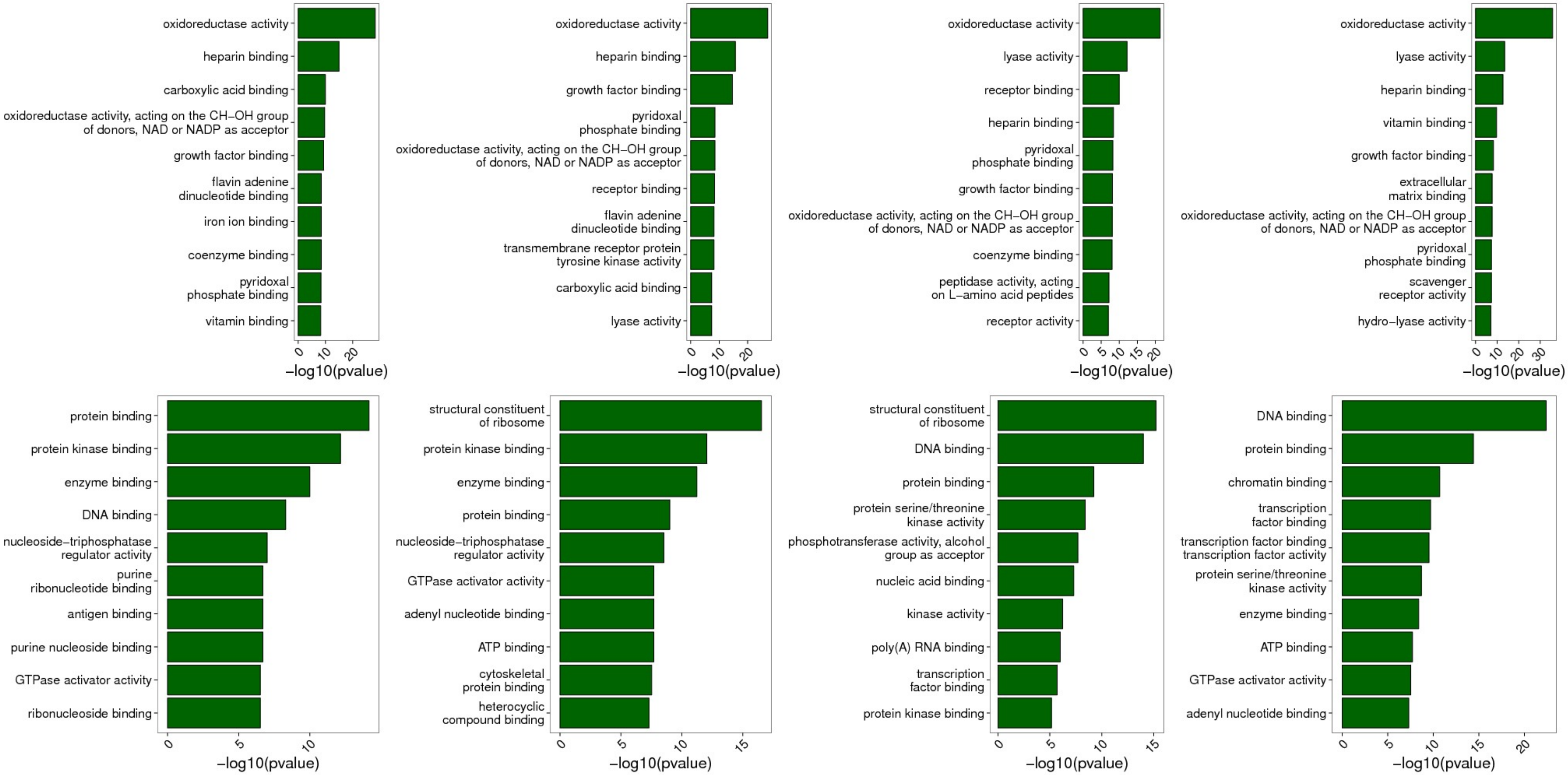

Figure S6: **Molecular Function GO term enrichment analysis for reference genes differentially expressed between liver and T cells.** This analysis was performed for each species individually (in columns: cattle, goat, chicken, pig in that order) and for genes over-expressed in liver (top) and over-expressed in T cells (bottom).

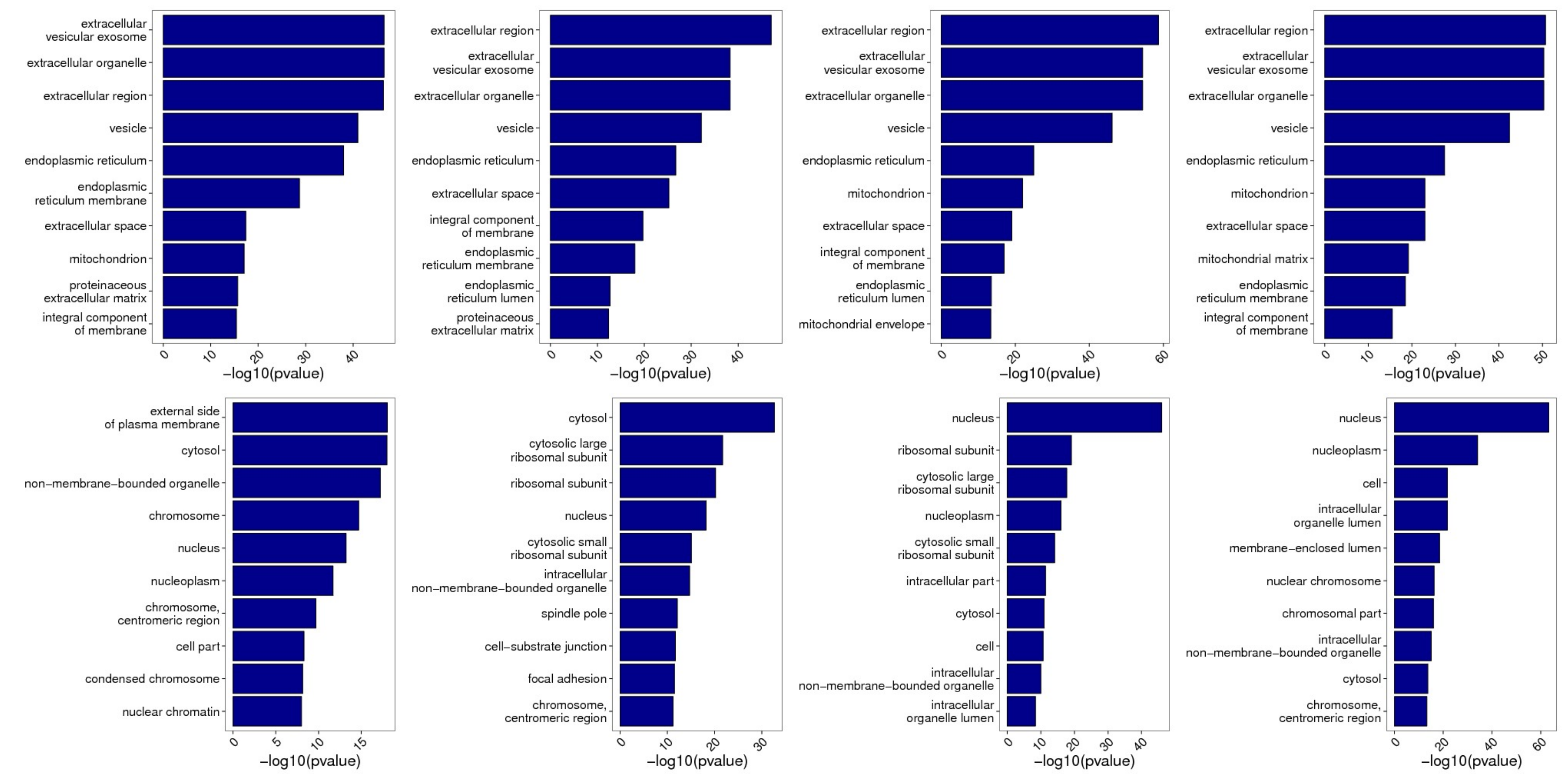

Figure S7: **Cellular Compartment GO term enrichment analysis for reference genes differentially expressed between liver and T cells.** This analysis was performed for each species individually (in columns: cattle, goat, chicken, pig in that order) and for genes over-expressed in liver (top) and over-expressed in T cells (bottom).

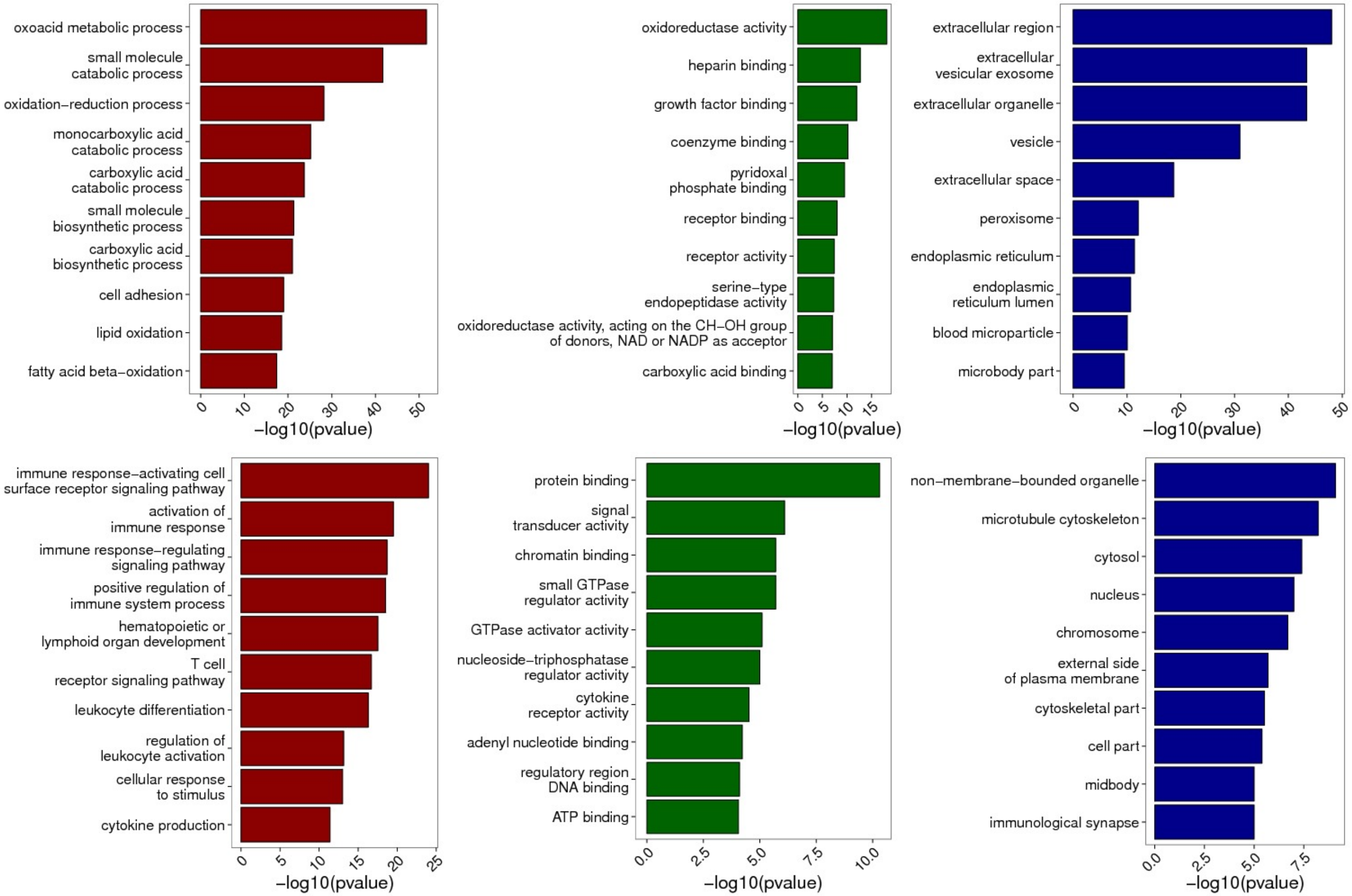

Figure S8: **GO term enrichment analysis for reference genes differentially expressed between liver and T cells in all species.** This analysis was performed for each GO (in column: Biological Process, Molecular Function, Cellular Compartment in that order) and for genes over-expressed in liver (top) and over-expressed in T cells (bottom).

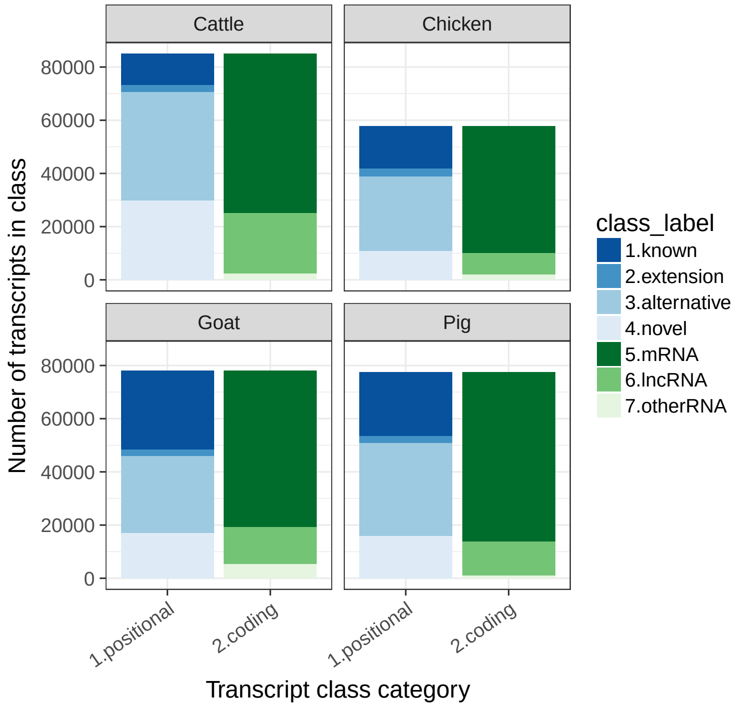

A

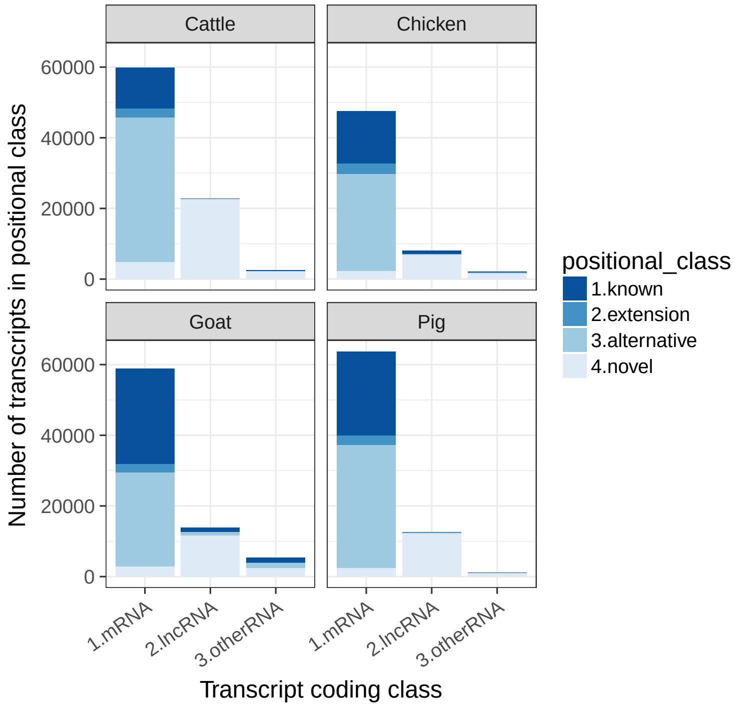

B

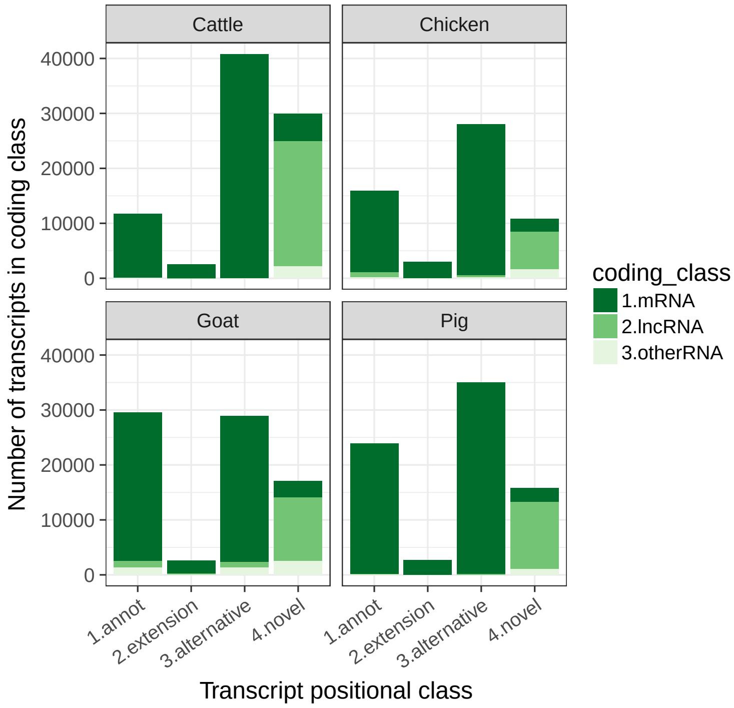

C

Figure S9: **Distribution of FR-AgENCODE transcripts into four positional (known, extension, alternative, novel) and three coding (mRNA, lncRNA, otherRNA) classes. (A)** Distribution of all FR-AgENCODE transcripts into positional and coding classes; **(B)** and **(C)** Distribution of FR-AgENCODE transcripts of each coding class into positional classes, and of each positional class into coding classes respectively.

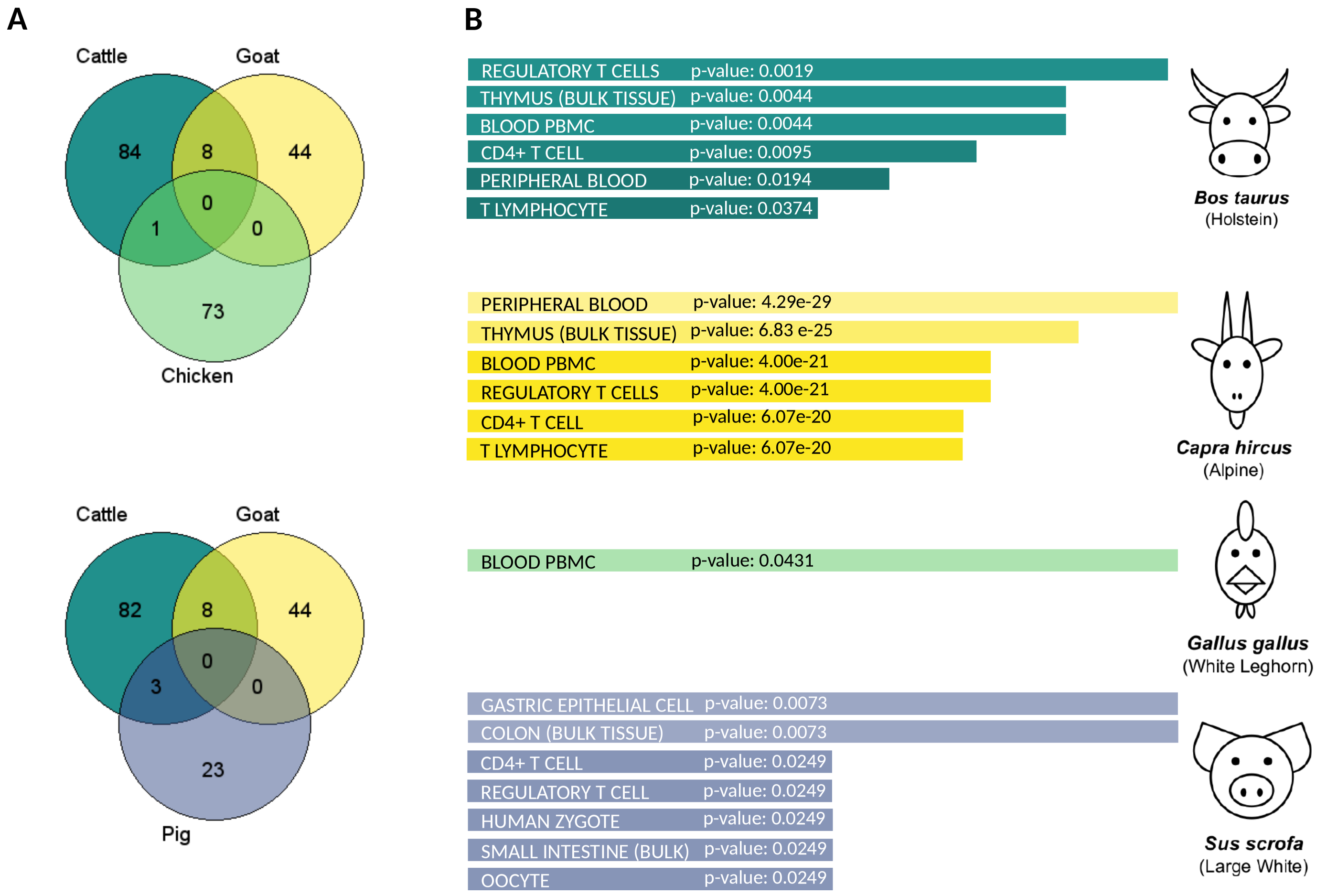

C

Novel coding FR-AgENCODE genes orthologous to human and shared by at least 2 livestock species
BIK
GCNA
TRBV20OR9-2
TRBV4-1
TRBV27
TRAV14DV4
TRAV12-3
TRAV17
TRBV3-1
TRBV25-1
INAFM2
FAM200B

Figure S10: **Novel coding FR-AgENCODE genes enrich the set of blood and T cell annotated genes.** (A) Venn diagrams of novel coding genes for each triplet of livestock species (two livestock genes are defined as orthologous if they project to the same human gene). (B) Gene set enrichment analysis on the human orthologs of the 93 cattle, 52 goat, 74 chicken and 26 pig genes, using EnrichR (Kuleshov et al. 2016; `http://amp.pharm.mssm.edu/Enrichr/`) on the ARCS4 Tissue dataset (`https://amp.pharm.mssm.edu/archs4/`). (C) Gene names of the 12 genes that are common to two livestock species. Out of these 12, 8 are coding for T cell Receptor Alpha or Beta Variable genes.

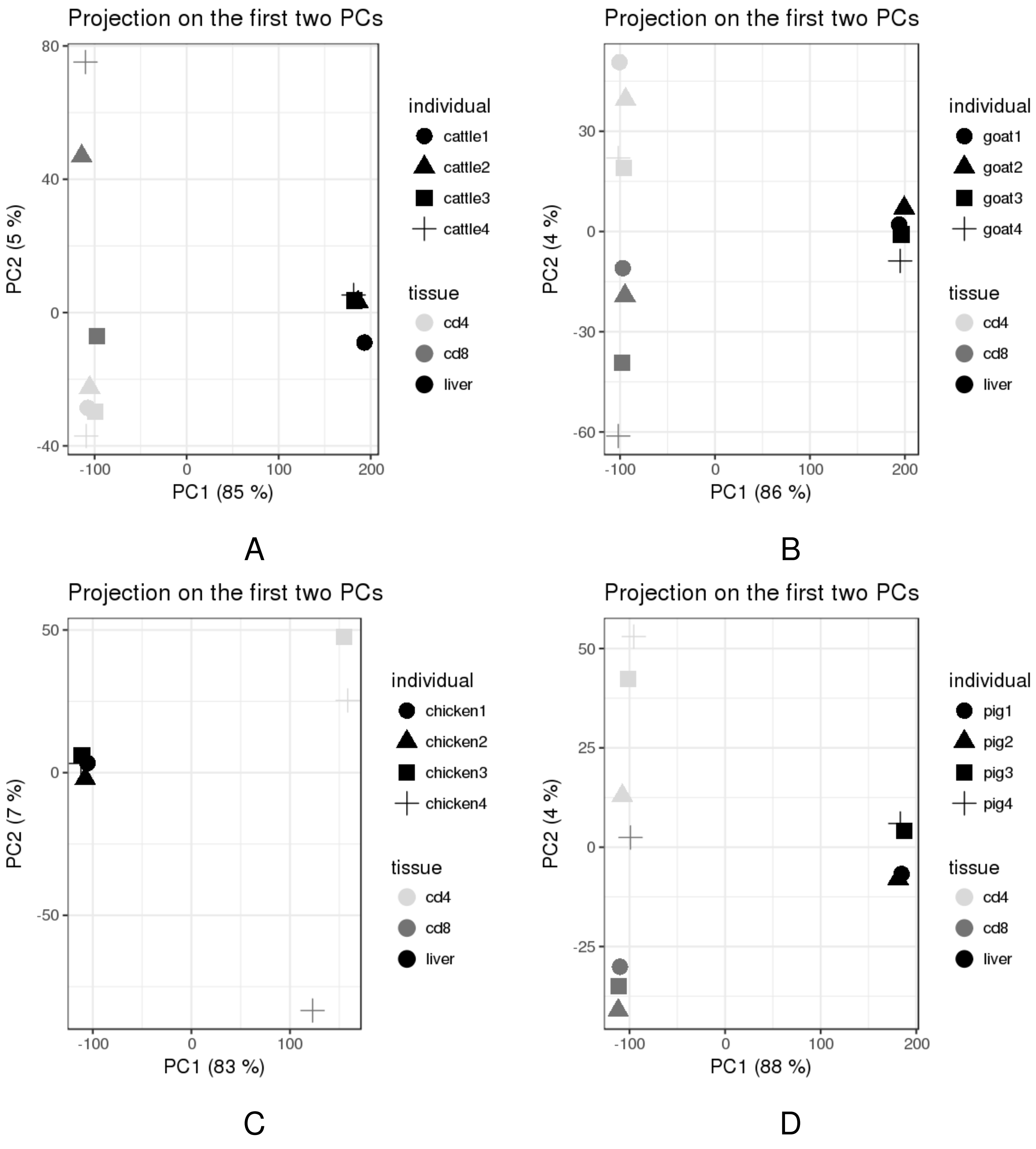

Figure S11: **RNA-seq sample PCA based on FR-AgENCODE gene expression.**

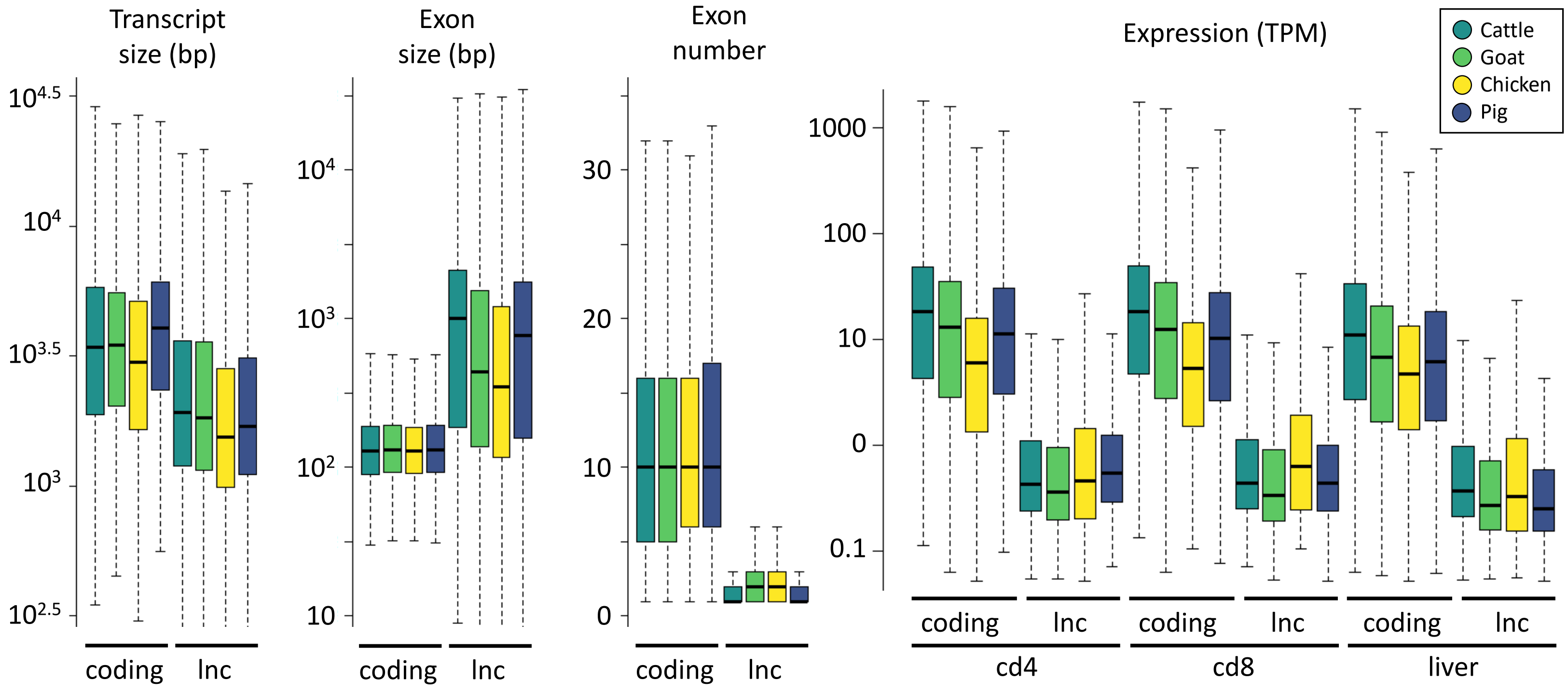

Figure S12: **LncRNA gene features.** Genomic structure features (left) and expression (right) are indicated for protein coding genes (coding) and lncRNA genes (lnc). The expression (TPM) is given for the three tissues and the four species; lncRNA genes are between 8- and 43-fold less expressed than protein coding genes (expression median: 0.4 vs. 10 TPM respectively).

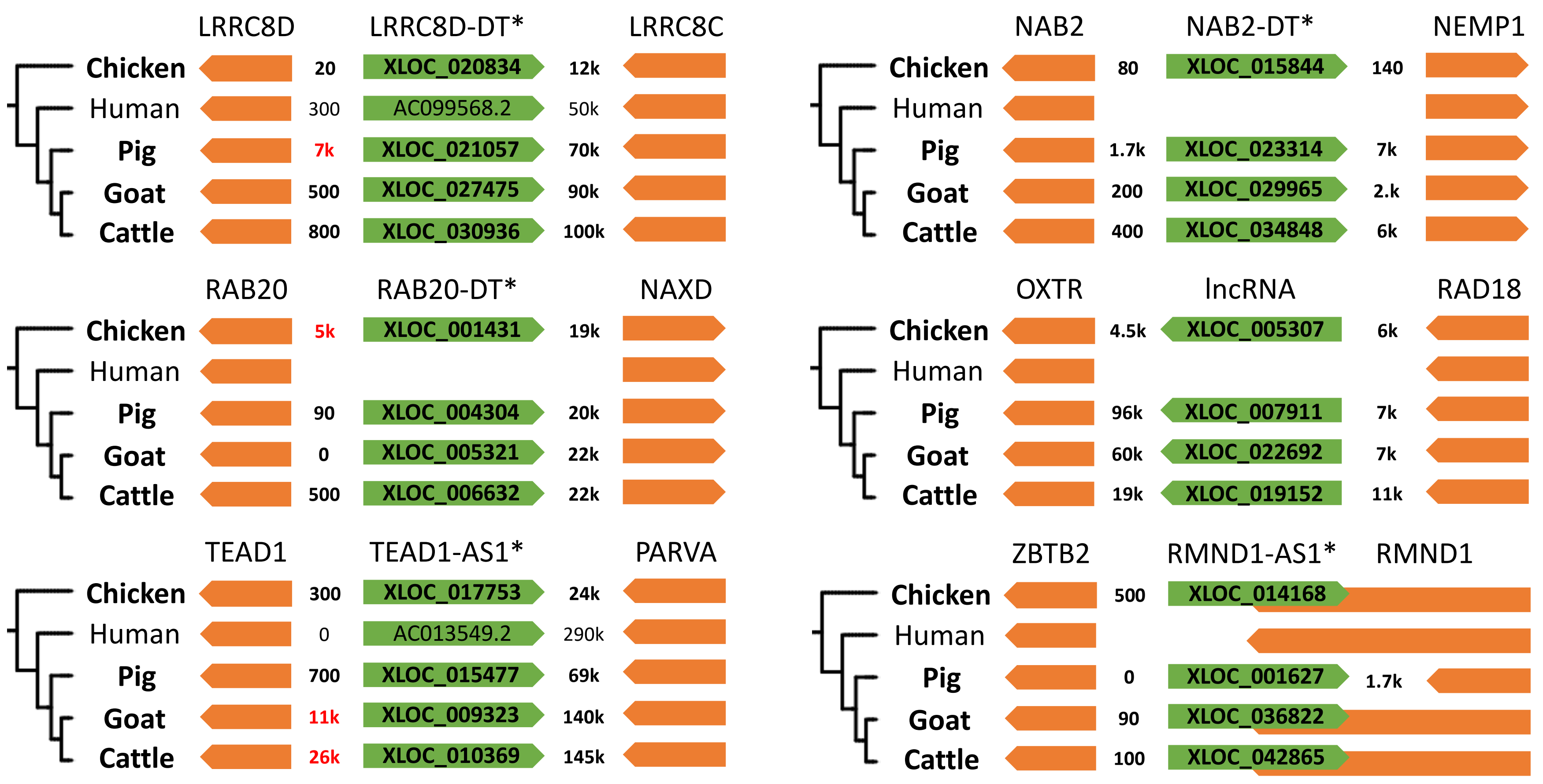

Figure S13: **Syntenic lncRNAs conserved between the 4 livestock species and human.** The 6 syntenic lncRNAs conserved in the 4 livestock species and human are represented in green and their surrounding protein coding genes in orange. Distances between the genes are indicated either in base pair or in kilo base pair (k). A distance of 0 means the genes are overlapping. A distance in red means a lower confidence in the orthology relationship for this species, after inspection of the distances found in the other species. When the lncRNA was not known before, a new gene name is proposed (asterisk).

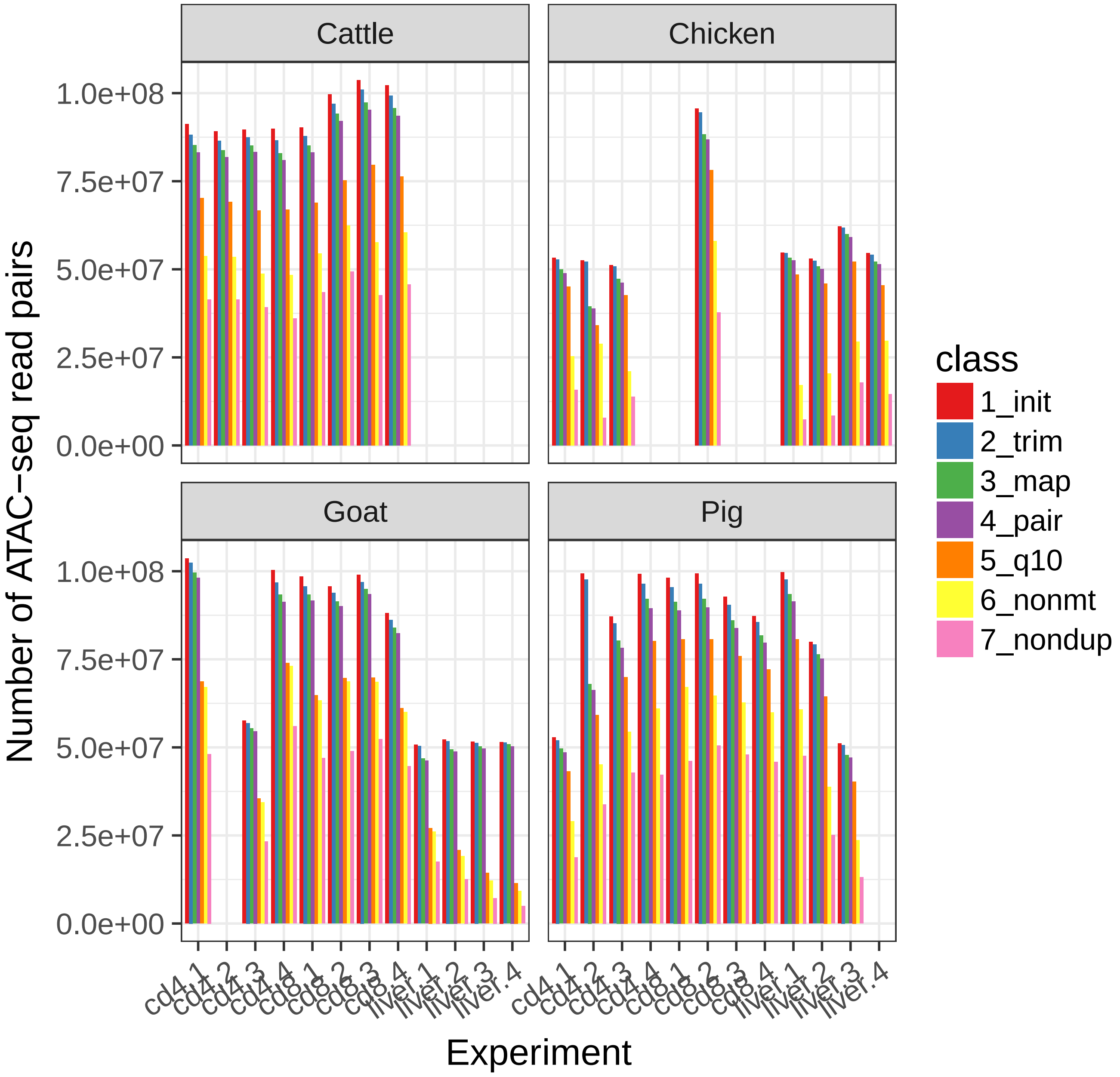

Figure S14: **ATAC-seq read pair summary statistics.** For each species and sample (labelled by its tissue and animal number), the number of initial read pairs (1_init), of read pairs obtained after trimming (2_trim), mapping (3_map), proper pairing (4_pair), q10 filtering (5_q10), mitochondrial read removal (6_nonmt), and PCR duplicate read removal (7_nondup) are shown.

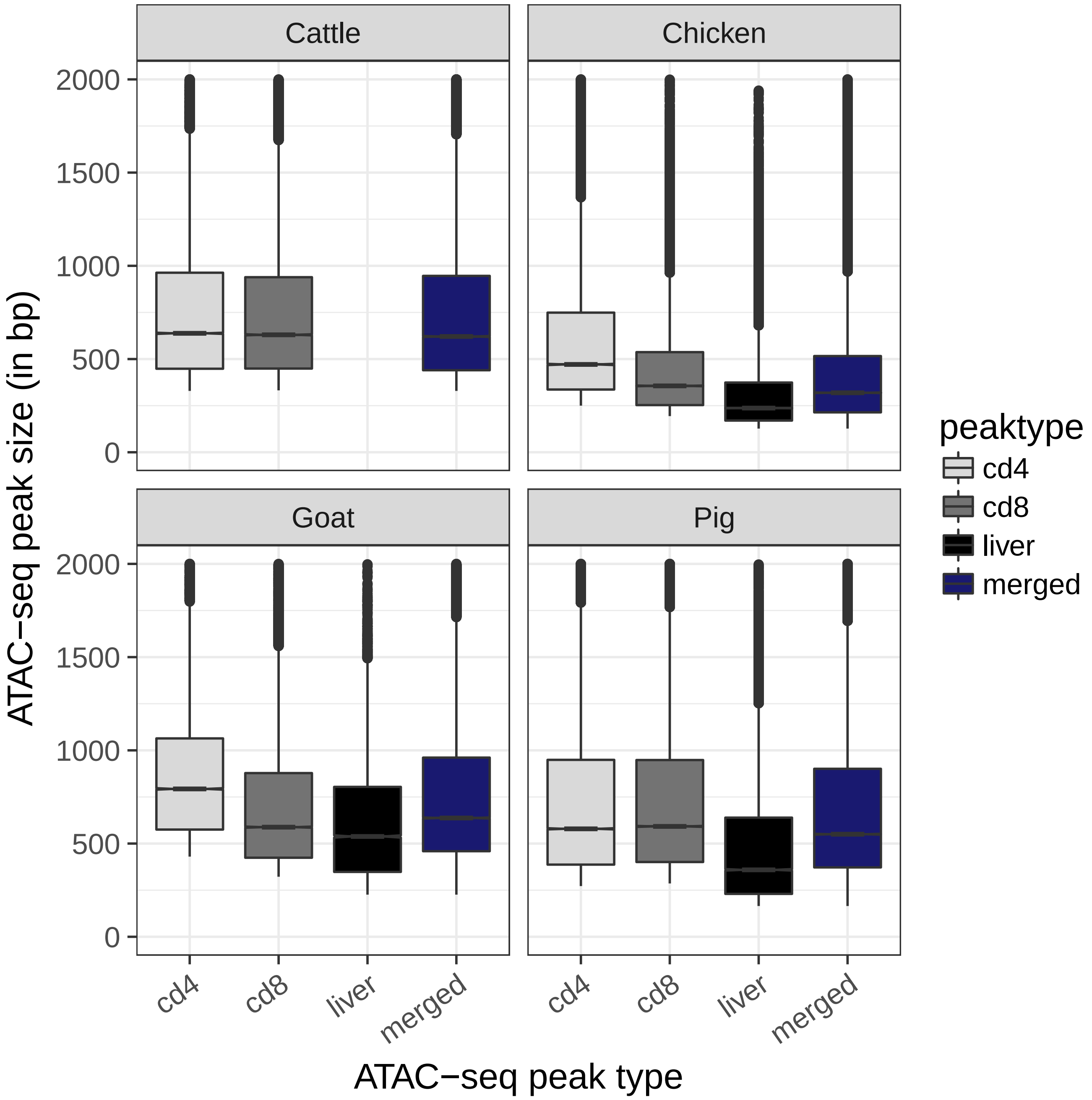

Figure S15: **ATAC-seq peak size distribution.** For each species, the size distribution of tissue and merged (i.e. across all tissues) ATAC-seq peaks are provided.

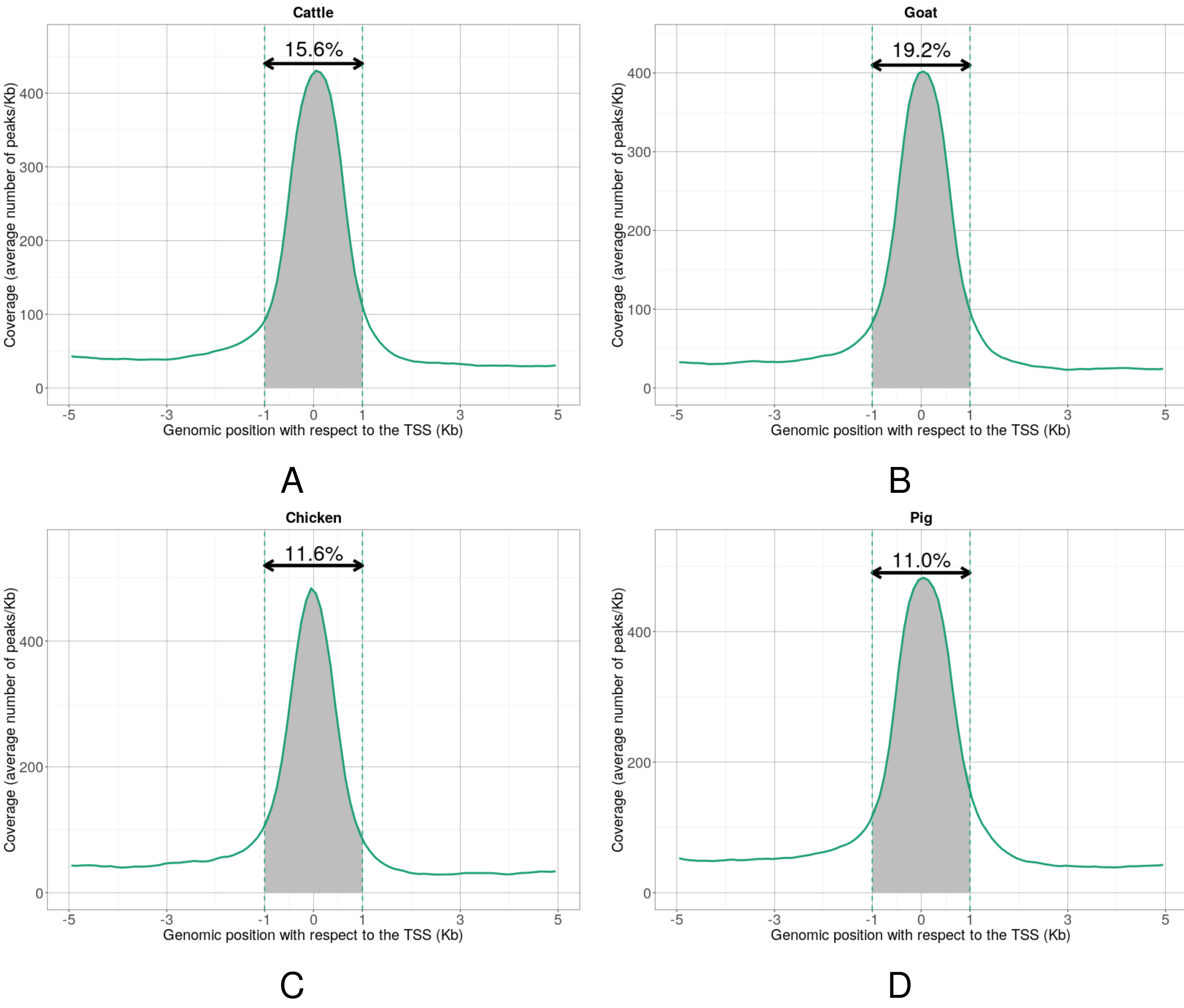

Figure S16: **Density of ATAC-seq peaks around starts of novel (i.e. not known) transcripts for cattle (A), goat (B), chicken (C) and pig (D)**

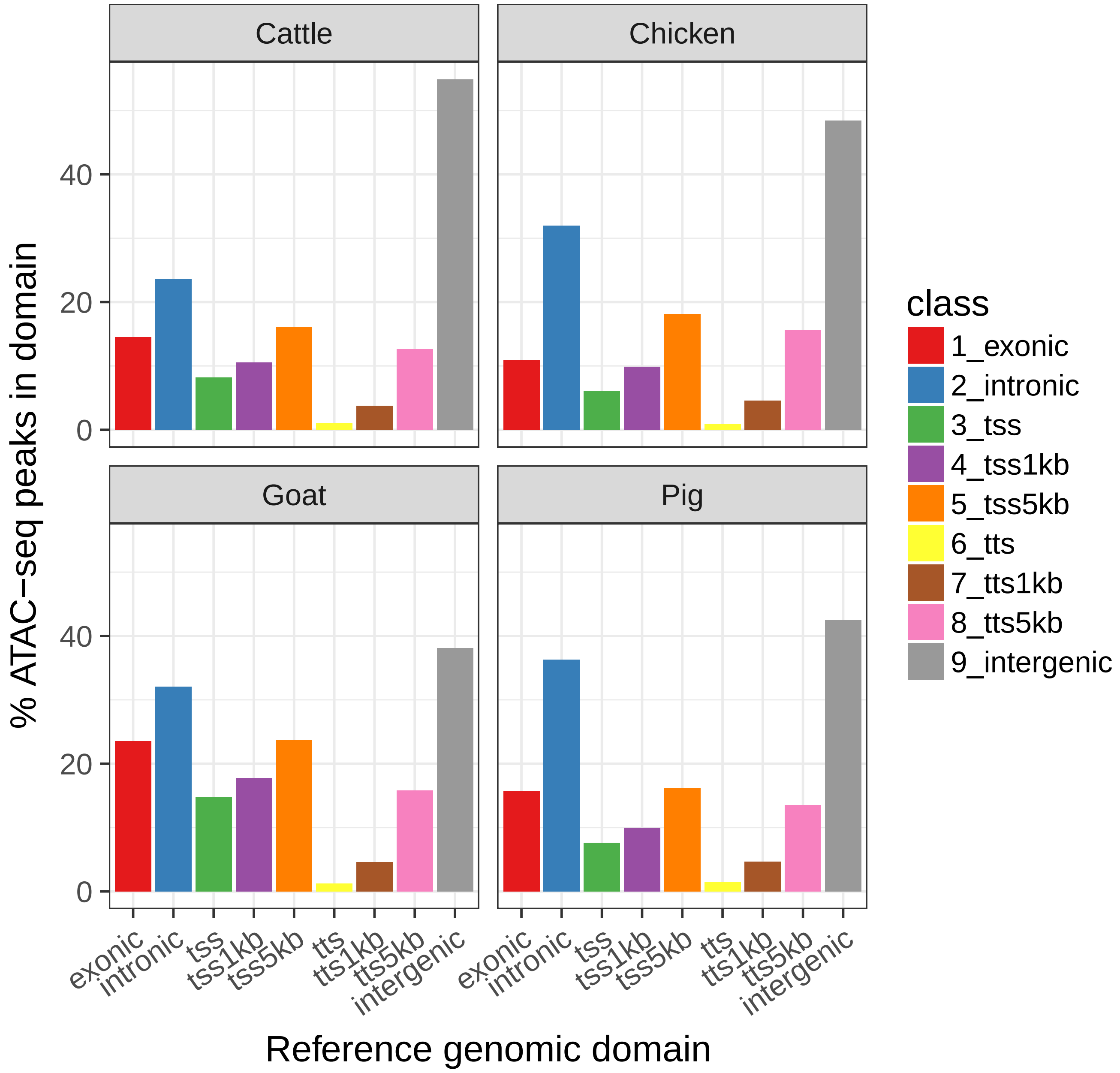

Figure S17: **ATAC-seq peak distribution into genomic domains.** For each species, are provided: the percentage of ATAC-seq peaks that are exonic, intronic, overlapping the TSS of a reference gene extended by 0, 1 or 5 Kb on each side, overlapping the TTS of a reference gene extended by 0, 1 or 5 Kb on each side and intergenic.

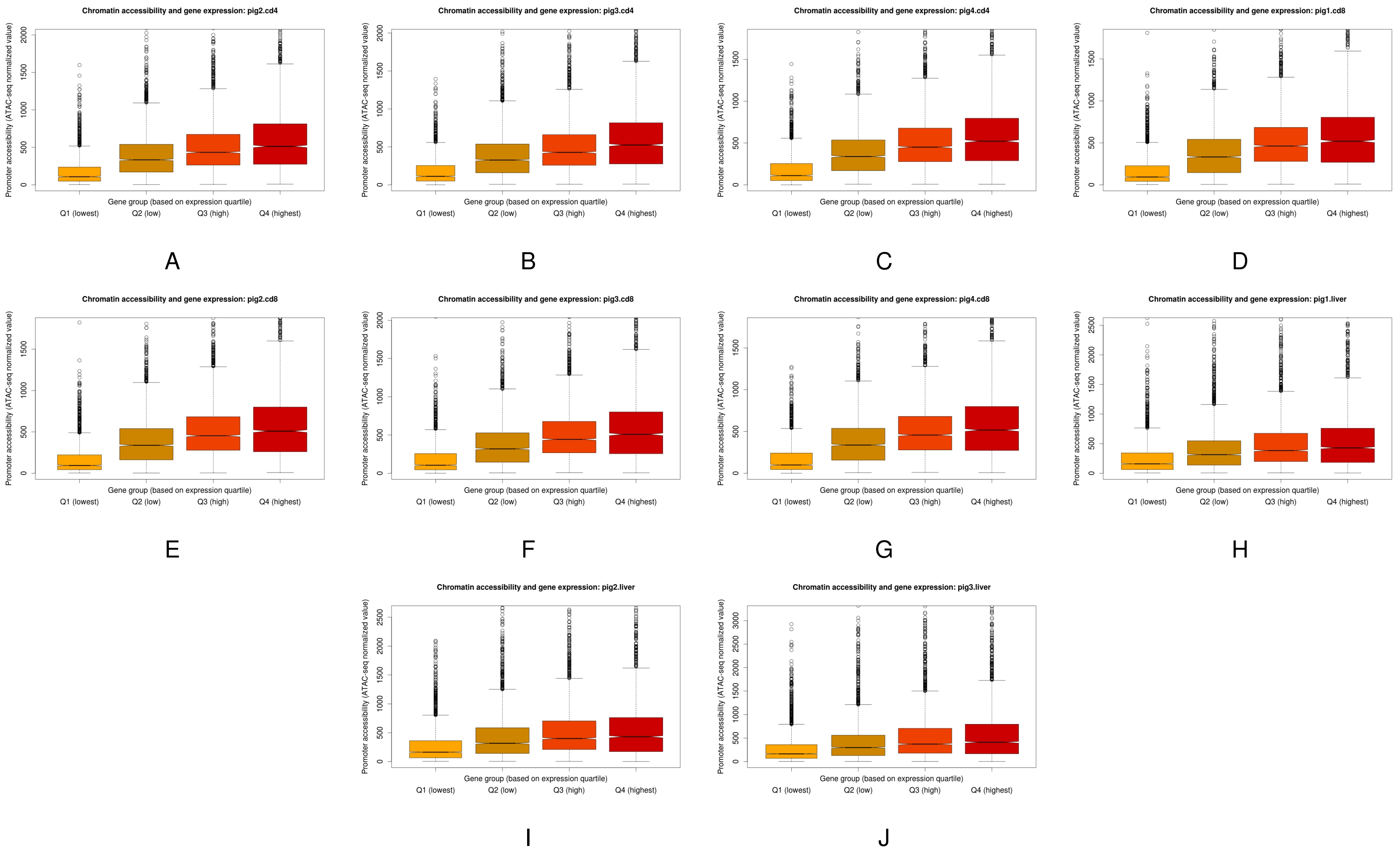

Figure S18: **Per sample promoter accessibility for four reference gene expression quartiles in pig.**

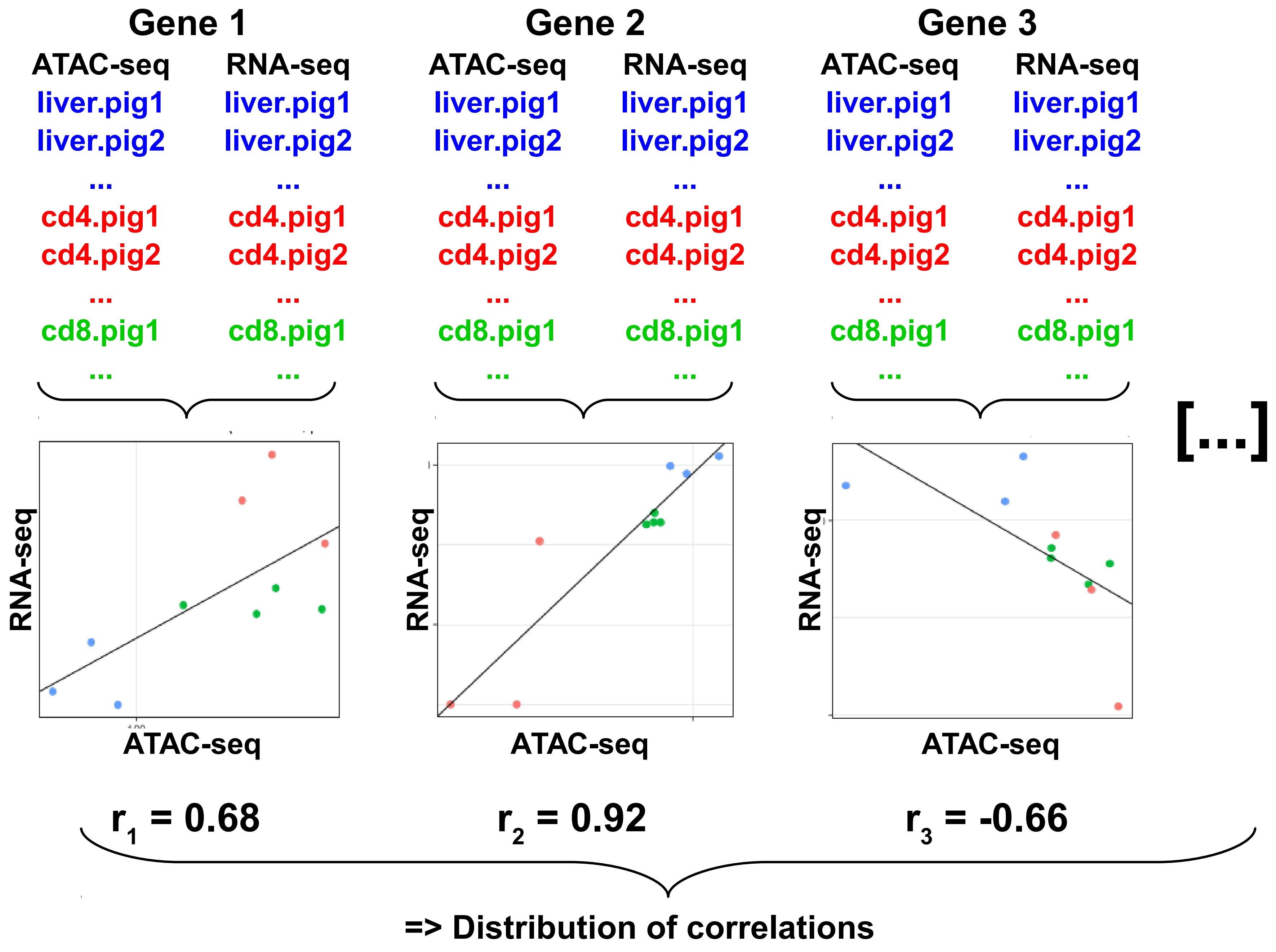

Figure S19: **Schematic illustration of the per-gene and cross-sample RNA-seq versus ATAC-seq correlation analysis.**

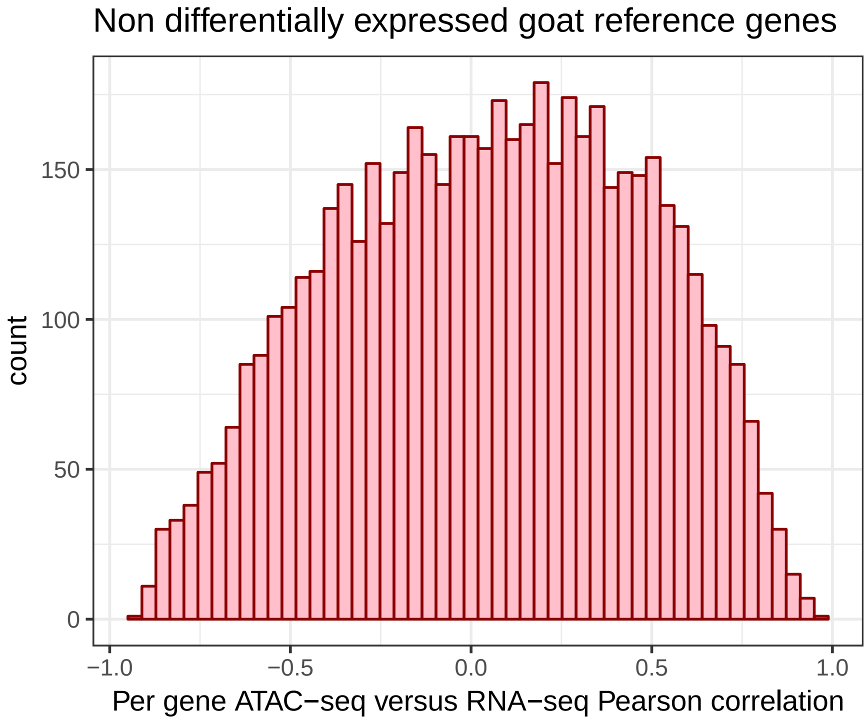

A

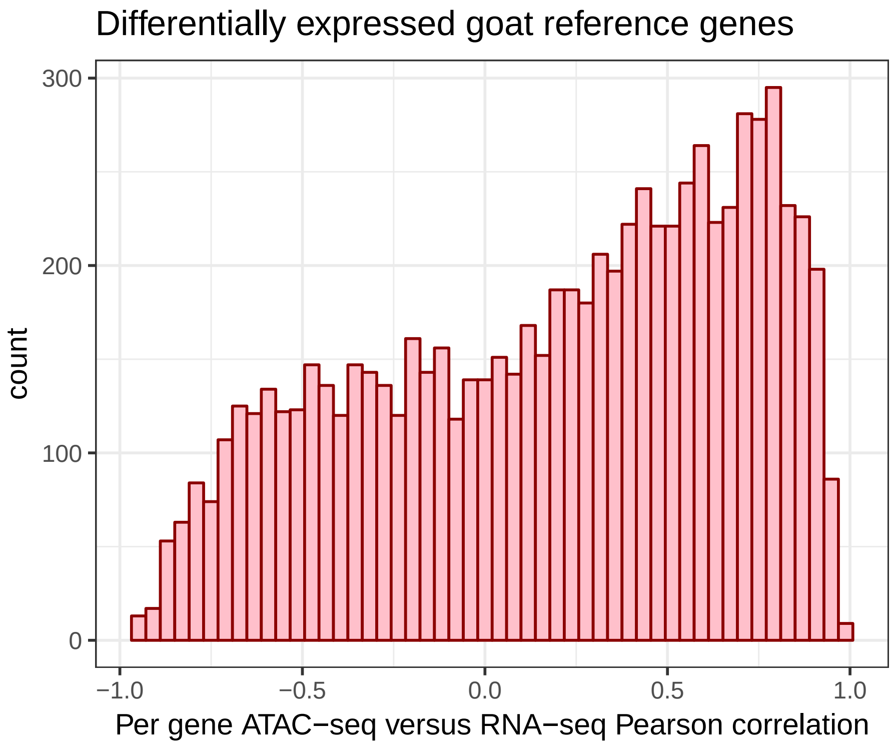

B

Figure S20: **Correlation between reference gene expression and chromatin accessibility at the TSS for goat.** For each pair of reference gene and ATAC-seq peak overlapping the reference gene TSS extended by 1 Kb on each side, the Pearson correlation was computed between the base 10 logarithm of the gene TMM and the base 10 logarithm of the normalized ATAC-seq signal at the peak. The distribution was then plotted for non DE genes **(A, top)** and for DE genes **(B, bottom)**.

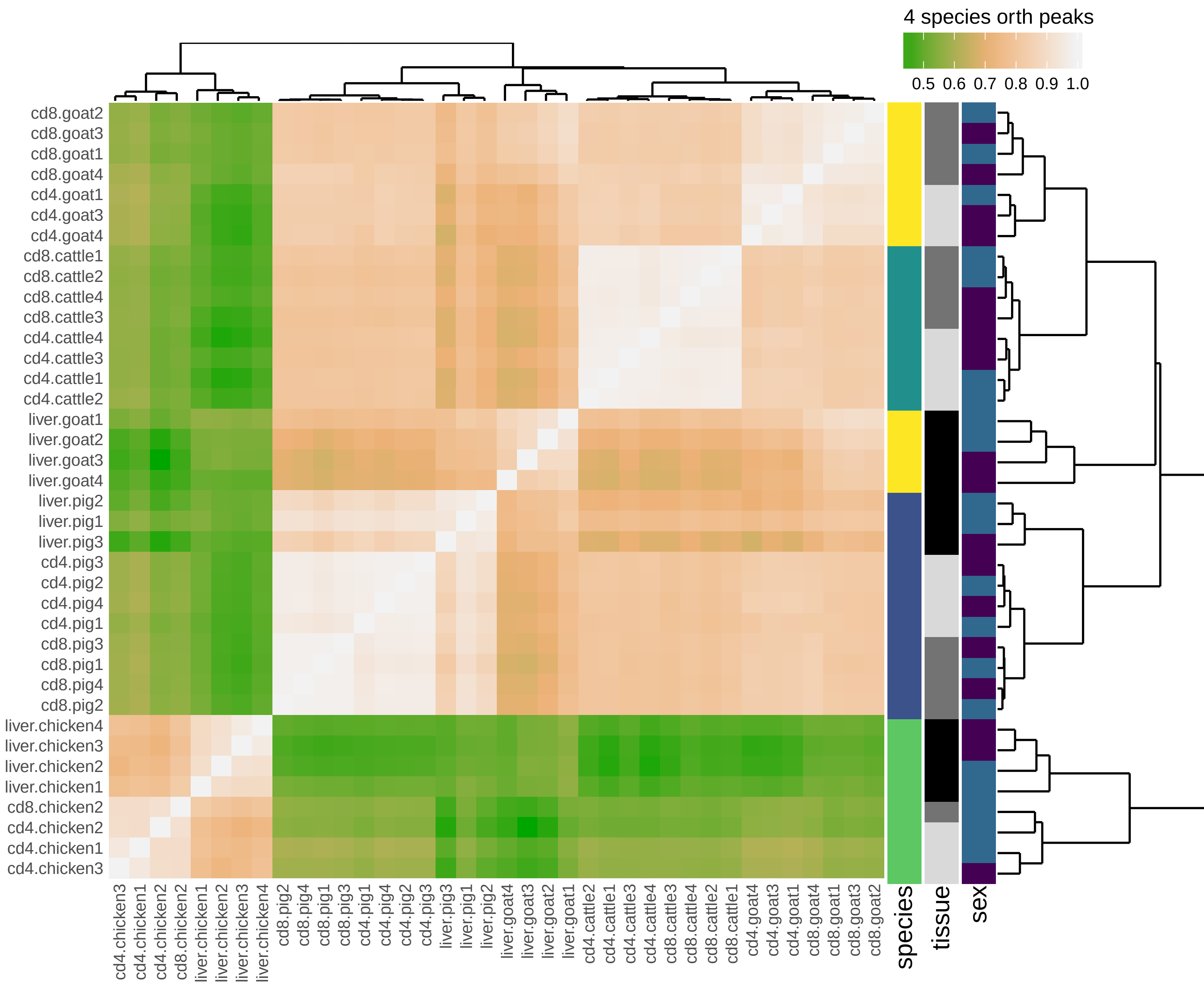

Figure S21: **ATAC-seq sample heatmap and hierarchical clustering based on the 1083 level 4 ATAC-seq peaks.** Pairwise similarity between samples is computed as the Pearson correlation between the base 10 logarithm of the normalized reads of the 1083 ATAC-seq peaks common to 4 species. These similarities are plotted as a heatmap, where samples appear both as rows and columns and are labelled by their species, tissue and the sex of the animal. The color of each heatmap cell also reflects the similarity (Pearson correlation) between each sample pair (the lighter, the higher). Hierarchical clustering is performed using one minus the squared Pearson correlation as a distance and the complete linkage aggregation method.

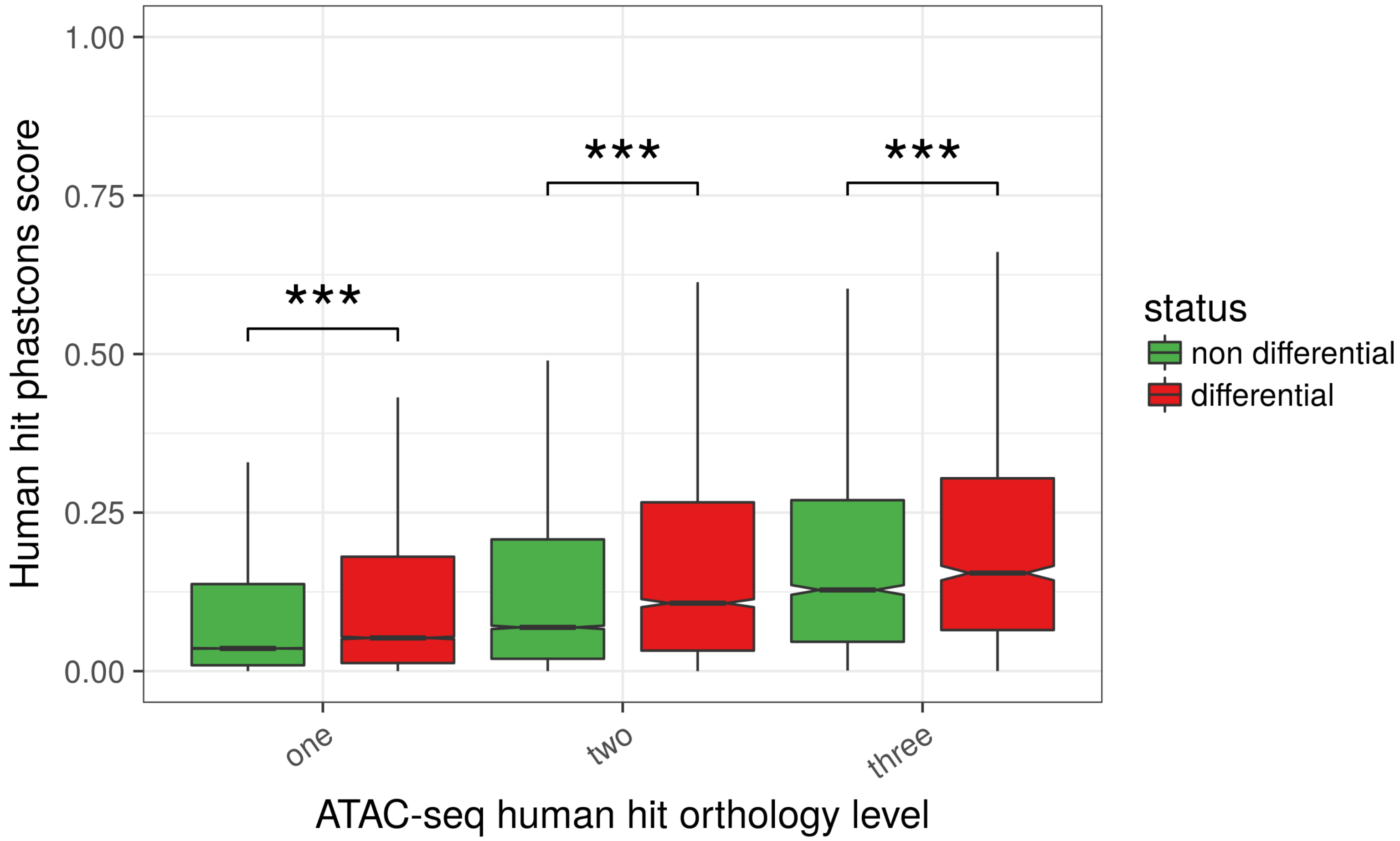

Figure S22: **Relation between non promoter proximal chromatin accessibility conservation and differential accessibility.** This figure is the same as main Figure 6 but is done restricting to ATAC-seq peaks not overlapping the TSS+-1kb in any of the species where it is present. Phastcons scores of these ATAC-seq peaks were plotted after dividing the human hits according to both their similarity level (between 1 and 3, x-axis, level 4 removed because there were less than 50 peaks in a category) and their differential accessibility (DA) status (DA in at least one species or DA in none of the 4 species, boxplot color). Although the phastcons score obviously increases with the similarity level, it is clear that, for a given similarity level, the phastcons score is higher for DA human hits than for non DA human hits (all similarity levels, p-values $< 1.2 \times 10^{-06}$ overall, Wilcoxon tests) (number of elements the boxplots from left to right: 151,783, 19,732, 11,922, 3,317, 2,100, 1,082).

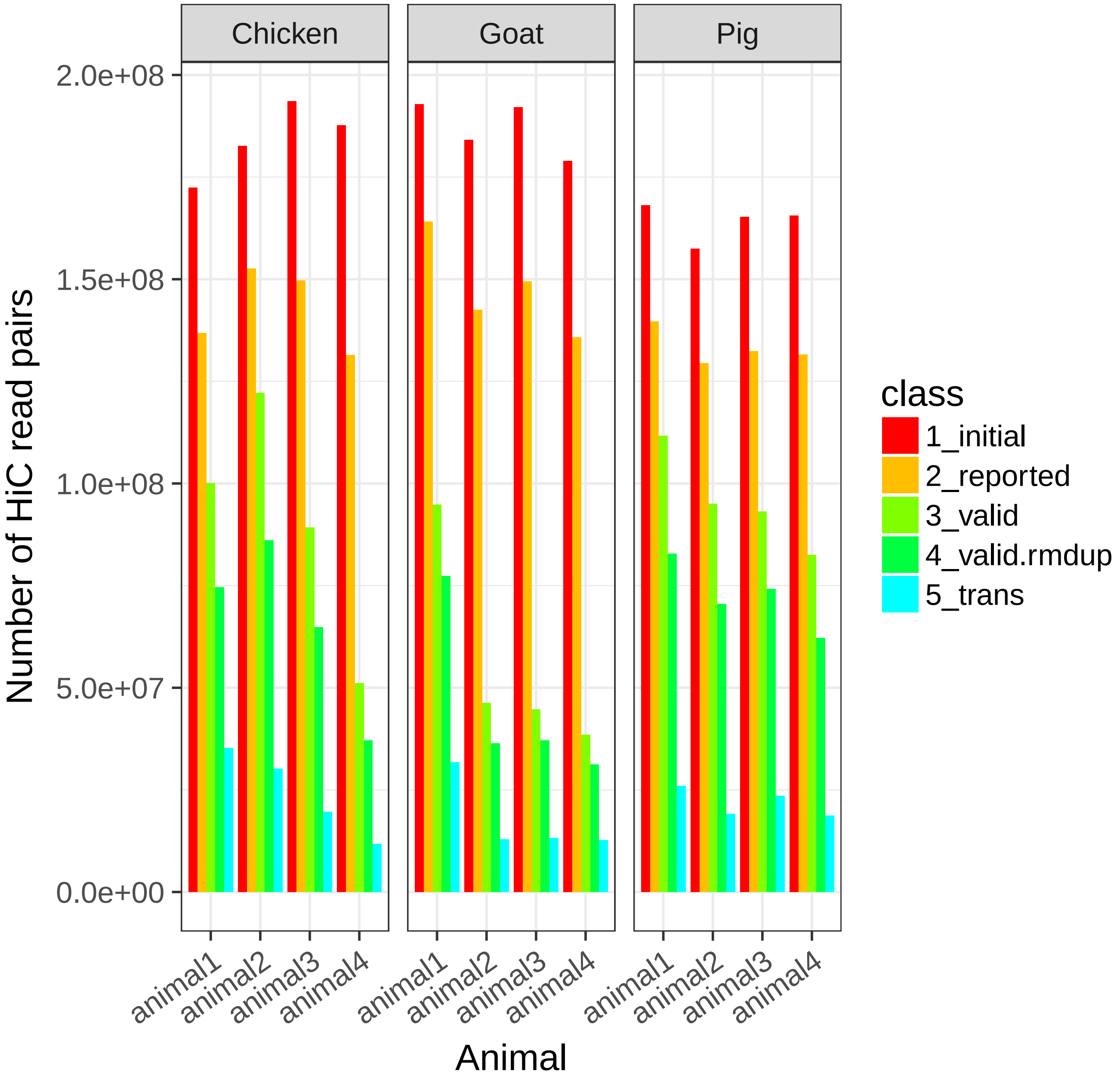

Figure S23: **Hi-C read summary statistics.** For each species and animal, are plotted the number of: initial read pairs after sequencing (1_initial), pairs with both reads mapped on the genome (2_reported), pairs in a valid configuration, that is when the sum of the distances from the reads to their next HindIII restriction sites downstream is comprised between 20bp and 1Kb (3_valid), pairs after removing PCR duplicates (4_valid_rmdup) and the number of read pairs supporting proximity between two different chromosomes (5_trans).

Identification of A/B compartments from Hi-C contact matrices

1. Merge the biological replicates by adding their read counts for each pair of bins

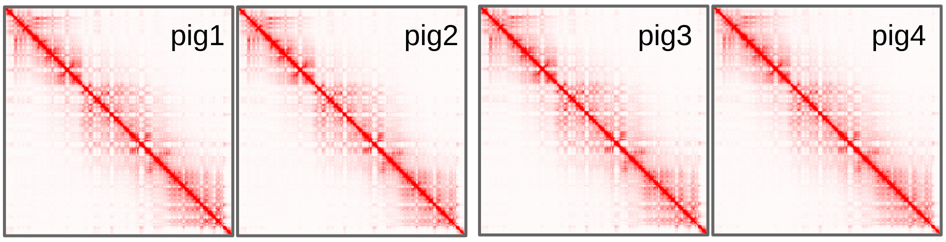

The Hi-C contact matrices from the 4 replicates (*Sus scrofa*, chromosome 1)

2. Normalization: matrix balancing and observed/expected (based on distance) (per chromosome)
3. Pearson correlation matrix from each pair of bins

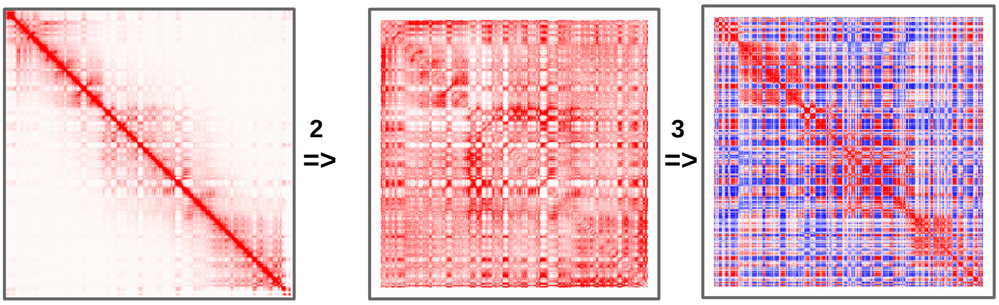

Raw matrix, normalized matrix and correlation matrix (*Sus scrofa*, chromosome 1)

3. Principal Component Analysis on the bins

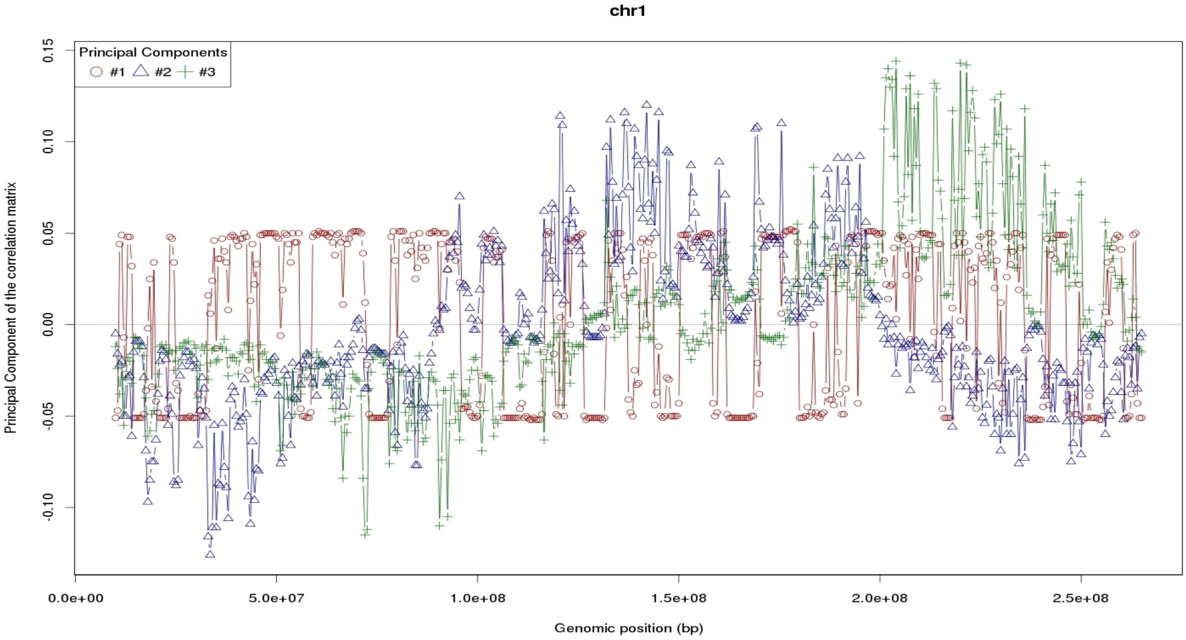

=> The sign of the 1st eigenvector (PC#1) defines the transitions between compartments.

Figure S24: **Method for predicting Hi-C A and B compartments.** Illustration of the A/B compartment calling workflow using the interaction matrices of the first chromosome in pig. Upper panels: interaction matrices at different steps of the workflow. Lower panel: the first three eigenvectors are shown along the chromosome to illustrate the relevance of PC#1 as the discriminative value to segregate bins between A and B compartments.

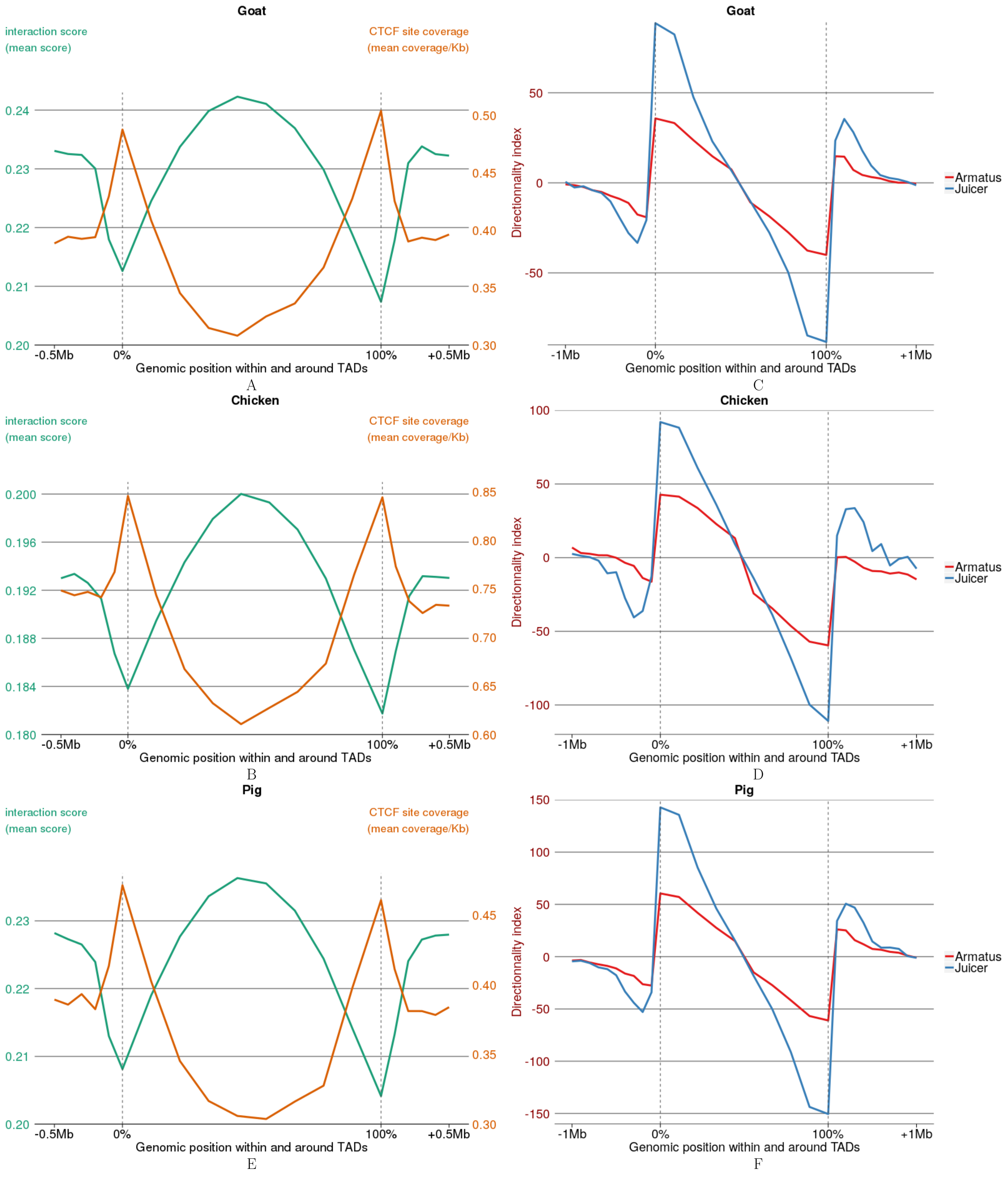

Figure S25: **CTCF motif density and local interaction score (left) and Directionality Index (DI, right) within and around Hi-C Armatus and Armatus/Juicer TADs.**

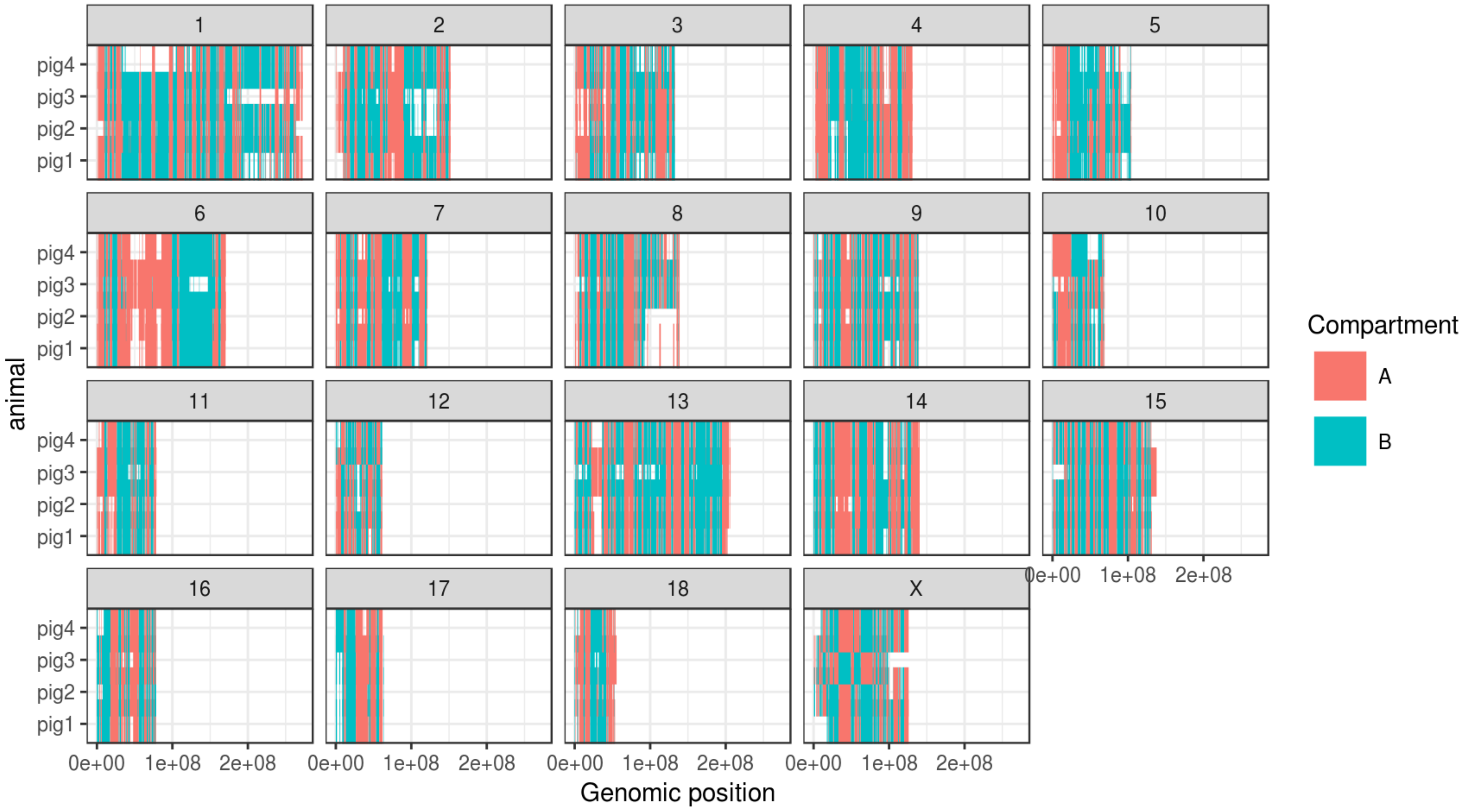

Figure S26: **Distribution of Hi-C A and B compartments along each chromosome and for each animal.** Genome-wide overview of compartment labels per 500Kb bin in pig for each animal. A general coherence can be observed across replicates. White regions are devoid of any called compartment.

Table S1: **Completed experiments.** Available data per animal, sample and experimental assay. Green: completed experiment. Red (NA): not available. Hi-C was attempted on liver samples only, and succeeded on all species but cattle. Consequently, ATAC-seq was not attempted on the cattle liver samples either.

RNA-seq

Cattle	cattle1	cattle2	cattle3	cattle4	Goat	goat1	goat2	goat3	goat4
cd4					cd4				
cd8	NA				cd8				
liver					liver				
**Chicken**	chicken1	chicken2	chicken3	chicken4	**Pig**	pig1	pig2	pig3	pig4
cd4	NA	NA			cd4	NA			
cd8	NA	NA	NA		cd8				
liver					liver				

ATAC-seq

Cattle	cattle1	cattle2	cattle3	cattle4	Goat	goat1	goat2	goat3	goat4
cd4					cd4		NA		
cd8					cd8				
liver	NA	NA	NA	NA	liver				
**Chicken**	chicken1	chicken2	chicken3	chicken4	**Pig**	pig1	pig2	pig3	pig4
cd4				NA	cd4				
cd8	NA		NA	NA	cd8				
liver					liver				NA

Hi-C

Cattle	cattle1	cattle2	cattle3	cattle4	Goat	goat1	goat2	goat3	goat4
liver	NA	NA	NA	NA	liver				
**Chicken**	chicken1	chicken2	chicken3	chicken4	**Pig**	pig1	pig2	pig3	pig4
liver					liver				

Table S2: **Genome and reference gene annotation used for each species.**

Species	Reference genome assembly				Reference gene annotation		
	Version	# fasta sequences	Size (in Gb)	Contig N50 (in Mb)	Source	# genes	# transcripts
Cattle	UMD3.1	3,317	2.67	0.1	Ensembl v90	24,616	26,740
Goat	CHIR_ARS1	29,907	2.92	26.2	NCBI vCHIR_ARS1	28,931	53,266
Chicken	GalGal5	23,475	1.23	2.9	Ensembl v90	24,881	38,118
Pig	Sscrofa11.1	613	2.5	48.2	Ensembl v90	25,880	49,448

Table S3: **Software used in the FR-AgENCODE project.**

Software name	Software version	Software URL
R	3.3.3	https://www.r-project.org/about.html
python	2.7.2	https://www.python.org/
samtools	1.3.1	http://www.htslib.org/
bedtools	2.26.0	http://bedtools.readthedocs.io/en/latest/
kentUtils	302.1.0	https://github.com/ENCODE-DCC/kentUtils
bwtool	Nov 2015 version	https://github.com/CRG-Barcelona/bwtool/wiki
emboss	6.4.0.0	http://emboss.sourceforge.net/
fastqc	0.11.2	https://www.bioinformatics.babraham.ac.uk/projects/fastqc/
cutadapt	1.8.3	http://cutadapt.readthedocs.io/en/stable/guide.html
trim_galore	0.4.0	https://www.bioinformatics.babraham.ac.uk/projects/trim_galore/
picardtools	2.1.1	https://broadinstitute.github.io/picard/
STAR	2.5.1b	https://github.com/alexdobin/STAR
cufflinks	2.2.1	http://cole-trapnell-lab.github.io/cufflinks/
RSEM	1.3.0	http://deweylab.biostat.wisc.edu/rsem/README.html
FEELnc	0.1.0	https://github.com/tderrien/FEELnc
Bowtie2	2.3.3.1	http://bowtie-bio.sourceforge.net/bowtie2/index.shtml
Macs2	2.1.1.20160309	https://chipster.csc.fi/manual/macs2.html
fimo	4.11.1	http://meme-suite.org/doc/fimo.html
HiC-Pro	2.9.0	http://nservant.github.io/HiC-Pro/
Armatus	2,1	https://www.cs.cmu.edu/~ckingsf/software/armatus/
HiTC	1.18.1	https://bioconductor.org/packages/release/bioc/html/HiTC.html
Juicer tools	1.5.3	https://github.com/theaidenlab/juicer/wiki/Juicer-Tools-Quick-Start
Last	956	http://last.cbrc.jp/

Table S4: **RNA-seq read mapping statistics.** Number and proportion of mapped and uniquely mapped RNA-seq read pairs for each species, replicate and tissue.

Species	Tissue	Animal	# read pairs	# read pairs mapped	% read pairs mapped	# uniquely mapped read pairs	% uniquely mapped read pairs (out of mapped)	% uniquely mapped read pairs (out of total)
Cattle	cd4	cattle1	115 398 649	111 206 516	96,4	108 830 546	97,9	94,3
	cd4	cattle2	118 918 186	114 474 132	96,3	112 127 763	98,0	94,3
	cd4	cattle3	121 389 996	113 765 363	93,7	108 717 357	95,6	89,6
	cd4	cattle4	116 083 510	112 298 028	96,7	110 019 926	98,0	94,8
	cd8	cattle2	117 869 348	113 617 513	96,4	111 375 385	98,0	94,5
	cd8	cattle3	118 910 270	114 458 706	96,3	111 966 825	97,8	94,2
	cd8	cattle4	121 883 485	117 905 219	96,7	115 423 917	97,9	94,7
	liver	cattle1	115 371 212	111 956 767	97,0	110 499 020	98,7	95,8
	liver	cattle2	119 870 689	116 728 118	97,4	115 092 798	98,6	96,0
	liver	cattle3	136 710 805	132 763 513	97,1	130 973 598	98,7	95,8
	liver	cattle4	118 889 544	115 841 946	97,4	114 125 584	98,5	96,0
Goat	cd4	goat1	133 365 348	130 269 121	97,7	123 559 681	94,8	92,6
	cd4	goat2	132 983 117	128 521 637	96,6	121 198 446	94,3	91,1
	cd4	goat3	133 831 464	129 566 423	96,8	122 990 885	94,9	91,9
	cd4	goat4	130 150 700	126 874 881	97,5	121 461 169	95,7	93,3
	cd8	goat1	130 970 787	127 546 631	97,4	120 904 297	94,8	92,3
	cd8	goat2	131 654 878	128 355 710	97,5	121 468 794	94,6	92,3
	cd8	goat3	132 923 610	129 276 688	97,3	123 235 599	95,3	92,7
	cd8	goat4	131 413 248	127 239 980	96,8	121 762 207	95,7	92,7
	liver	goat1	112 623 152	110 432 315	98,1	105 943 818	95,9	94,1
	liver	goat2	120 492 856	117 992 580	97,9	112 453 956	95,3	93,3
	liver	goat3	133 931 176	131 150 414	97,9	126 458 023	96,4	94,4
	liver	goat4	125 330 385	122 259 872	97,6	118 111 570	96,6	94,2
Chicken	cd4	chicken3	91 907 306	86 645 024	94,3	83 374 510	96,2	90,7
	cd4	chicken4	90 843 387	79 772 579	87,8	76 778 803	96,2	84,5
	cd8	chicken4	90 476 514	82 431 332	91,1	79 685 312	96,7	88,1
	liver	chicken1	119 681 527	114 944 560	96,0	111 122 525	96,7	92,8
	liver	chicken2	107 777 068	103 770 521	96,3	100 787 680	97,1	93,5
	liver	chicken3	121 616 481	116 940 968	96,2	113 315 177	96,9	93,2
	liver	chicken4	124 089 440	119 195 187	96,1	115 442 821	96,9	93,0
Pig	cd4	pig2	116 273 646	113 997 546	98,0	109 051 735	95,7	93,8
	cd4	pig3	118 260 498	115 042 891	97,3	110 755 493	96,3	93,7
	cd4	pig4	114 159 672	111 934 574	98,1	108 379 228	96,8	94,9
	cd8	pig1	231 339 734	225 402 022	97,4	219 199 568	97,2	94,8
	cd8	pig2	116 383 341	114 513 826	98,4	110 146 147	96,2	94,6
	cd8	pig3	116 696 412	113 825 826	97,5	110 613 640	97,2	94,8
	cd8	pig4	117 449 531	114 751 230	97,7	108 598 741	94,6	92,5
	liver	pig1	116 242 745	113 660 238	97,8	110 849 470	97,5	95,4
	liver	pig2	117 818 284	115 364 464	97,9	111 627 222	96,8	94,7
	liver	pig3	110 567 402	108 214 816	97,9	105 590 955	97,6	95,5
	liver	pig4	114 769 769	112 137 997	97,7	109 296 648	97,5	95,2

Table S5: **Differentially Expressed (DE) reference genes.** Number of differentially expressed reference genes obtained by the two statistical models (see main text and Methods)

Differential analysis model	Tissue 1	Tissue 2	log(Tissue2 / Tissue1)	Number of DE reference genes			
				Cattle	Goat	Chicken	Pig
1 (tissue pairs)	cd4	cd8	>0	751	1098	1 504	867
	cd4	cd8	<0	463	748	592	604
	cd4	liver	>0	5048	6303	5 112	5 725
	cd4	liver	<0	3887	4304	3 351	3 653
	cd8	liver	>0	4890	6134	4 146	5 665
	cd8	liver	<0	3911	4397	3 087	3 792
2 (tcell vs liver)	cd	liver	>0	4992	6188	4 307	5 666
	cd	liver	<0	3943	4384	2 640	3 772

Table S6: **Genes consistently over-expressed in CD4+ compared to CD8+ or reciprocally, in four livestock species.** For the 39 genes consistently seen as over-expressed in CD4+ with respect to CD8+ (10 genes) or reciprocally (29 genes), are indicated: the human gene ID, the gene name in a column that indicates the cell type in which the gene is over-expressed, the TPM in human CD4+ and CD8+ cells from the Blueprint project (CD4-positive, alpha-beta T cell and CD8-positive, alpha-beta T cell respectively), whether or not these expression levels are consistent with the differential behavior observed in livestock, the expression in livestock CD4+ and CD8+ (average across samples) and a reference describing the role of the gene in blood cells when available.

Human gene id	Gene name (in column corresponding to over-expression)		TPM in human		Consistency between livestock and human (FC>1)	TPM in cattle		TPM in goat		TPM in chicken		TPM in pig	
	CD4+	CD8+	CD4+	CD8+		CD4+	CD8+	CD4+	CD8+	CD4+	CD8+	CD4+	CD8+
ENSG00000102245 [1]	CD40LG	.	27.0	0.5	yes	97.9	7.6	51.6	10.5	164.8	7.5	153.4	28.7
ENSG00000106537	TSPAN13	.	0.4	0.5	no	118.8	62.6	93.9	32.6	4.0	0.5	3.4	1.0
ENSG00000107485 [2]	GATA3	.	6.0	4.0	yes	37.7	11.5	43.3	18.8	113.5	7.6	51.1	30.0
ENSG00000115602	IL1RL1	.	NA	0.1	NA	4.7	0.3	5.2	2.2	5.5	0.2	4.2	1.0
ENSG00000126353 [3]	CCR7	.	312.0	102.0	yes	33.7	11.3	164.5	44.6	96.2	21.7	87.1	33.2
ENSG00000130396	AFDN	.	0.9	0.3	yes	6.6	2.9	17.8	2.8	3.3	0.8	2.4	0.1
ENSG00000163599 [4]	CTLA4	.	39.0	2.0	yes	6.1	0.6	19.8	1.4	635.6	16.9	15.0	2.2
ENSG00000178199	ZC3H12D	.	17.0	6.0	yes	30.5	13.6	45.7	9.4	1,384.7	134.7	26.3	4.5
ENSG00000178562 [5]	CD28	.	161.0	74.0	yes	34.4	9.3	91.5	11.0	208.3	14.8	107.4	27.6
ENSG00000178573 [6]	MAF	.	3.0	1.0	yes	13.2	1.5	11.8	5.4	6.8	0.3	15.0	2.5
ENSG00000139567	.	ACVRL1	NA	NA	NA	5.9	48.0	5.0	18.9	5.2	10.1	0.0	0.2
ENSG00000169252 [7]	.	ADRB2	0.6	2.0	yes	3.9	17.9	0.6	7.3	57.8	219.3	2.7	15.2
ENSG00000156966	.	B3GNT7	0.1	0.2	yes	1.7	7.2	0.2	0.7	0.0	2.8	0.0	1.4
ENSG00000136573	.	BLK	1.0	4.0	yes	15.9	27.0	3.8	53.2	3.9	15.4	1.5	32.8
ENSG00000182985	.	CADM1	0.2	NA	NA	4.1	21.4	1.1	3.5	1.6	3.9	0.0	0.2
ENSG00000271503 [8]	.	CCL5	8.0	38.0	yes	33.0	1,205.8	16.2	2,009.6	1.2	102.1	190.2	2,465.9
ENSG00000172116 [9]	.	CD8B	2.0	210.0	yes	9.7	260.7	17.6	514.5	0.8	173.0	1.1	115.4
ENSG00000109943 [10]	.	CRTAM	3.0	54.0	yes	2.3	16.3	4.0	16.2	0.8	52.0	3.1	37.6
ENSG00000100592	.	DAAM1	4.0	3.0	no	5.3	5.0	2.1	2.8	0.3	0.3	5.5	5.7
ENSG00000035664 [11]	.	DAPK2	0.3	0.9	yes	1.8	8.2	0.9	10.1	3.0	11.7	14.9	60.1
ENSG00000213853	.	EMP2	0.6	0.4	no	0.1	0.7	1.9	6.9	0.1	0.5	0.1	0.9
ENSG00000163508 [12]	.	EOMES	0.9	1.0	yes	1.0	11.3	1.0	17.5	2.9	133.4	1.0	12.5
ENSG00000139132	.	FGD4	0.2	2.0	yes	3.3	8.5	0.9	2.3	0.9	6.8	0.2	5.3
ENSG00000160219	.	GAB3	4.0	4.0	no	3.3	8.6	9.2	25.1	4.4	38.4	8.9	27.2
ENSG00000125245	.	GPR18	17.0	26.0	yes	7.8	22.8	9.0	22.8	13.5	104.8	39.0	297.6
ENSG00000100385 [13]	.	IL2RB	7.0	9.0	yes	42.7	131.6	134.9	573.8	30.2	190.7	62.6	205.2
ENSG00000157404	.	KIT	0.9	2.0	yes	2.4	7.9	2.4	6.4	0.1	0.3	0.0	1.2
ENSG00000086730 [14]	.	LAT2	0.8	4.0	yes	46.1	156.3	20.0	46.2	2.0	11.9	22.1	123.7
ENSG00000189067	.	LITAF	68.0	58.0	no	24.0	98.1	26.0	82.3	12.3	41.5	4.6	21.4
ENSG00000158186	.	MRAS	0.1	NA	NA	0.9	5.9	2.1	6.4	16.4	32.9	0.0	0.0
ENSG00000100311	.	PDGFB	0.9	NA	NA	6.1	25.8	2.2	8.6	0.2	0.9	0.7	5.6
ENSG00000115956	.	PLEK	2.0	22.0	yes	10.4	32.1	9.1	52.4	36.8	43.0	5.2	55.9
ENSG00000141956	.	PRDM15	5.0	2.0	no	1.8	1.8	3.4	3.0	7.6	3.3	3.4	3.4
ENSG00000198915	.	RASGEF1A	0.1	0.1	no	3.3	8.3	2.5	10.8	27.6	61.8	0.3	3.0
ENSG00000119729	.	RHOQ	6.0	4.0	no	18.1	74.3	14.5	45.9	0.6	8.9	2.9	16.5
ENSG00000136158 [15]	.	SPRY2	NA	1.0	yes	1.0	23.5	0.6	11.3	2.0	66.0	0.1	40.5
ENSG00000204634	.	TBC1D8	0.6	2.0	yes	12.6	62.9	5.8	17.0	0.3	0.6	0.2	0.5
ENSG00000070759	.	TESK2	6.0	6.0	no	4.8	15.2	6.9	29.1	3.3	9.5	5.6	39.0
ENSG00000165914	.	TTC7B	0.4	0.5	yes	0.4	2.0	0.2	0.7	1.0	2.5	0.1	0.6

[1] Blotta MH, Marshall JD, DeKruyff RH, Umetsu DT. Cross-linking of the CD40 ligand on human CD4+ T lymphocytes generates a costimulatory signal that up-regulates IL-4 synthesis. The Journal of Immunology. 1996 May 1;156(9):3133-40.
[2] Tindemans I, Serafini N, Di Santo JP, Hendriks RW. GATA-3 function in innate and adaptive immunity. Immunity. 2014 Aug 21;41(2):191-206.
[3] Förster R, Davalos-Misslitz AC, Rot A. CCR7 and its ligands: balancing immunity and tolerance. Nature Reviews Immunology. 2008 May;8(5):362.
[4] Zemmour D, Zilionis R, Kiner E, Klein AM, Mathis D, Benoist C. Single-cell gene expression reveals a landscape of regulatory T cell phenotypes shaped by the TCR. Nature immunology. 2018 Mar;19(3):291.
[5] Esensten JH, Helou YA, Chopra G, Weiss A, Bluestone JA. CD28 costimulation: from mechanism to therapy. Immunity. 2016 May 17;44(5):973-88.
[6] Wheaton JD, Yeh CH, Ciofani M. Cutting Edge: c-Maf Is Required for Regulatory T Cells To Adopt RORγt+ and Follicular Phenotypes. The Journal of Immunology. 2017 Dec 15;199(12):3931-6.
[7] Landmann R. Beta-adrenergic receptors in human leukocyte subpopulations. European journal of clinical investigation. 1992 Oct;22:30-6.
[8] DeVico AL, Gallo RC. Control of HIV-1 infection by soluble factors of the immune response. Nature Reviews Microbiology. 2004 May;2(5):401.
[9] Kioussis D, Ellmeier W. Decision making in the immune system: Chromatin and CD4, CD8A and CD8B gene expression during thymic differentiation. Nature Reviews Immunology. 2002 Dec;2(12):909.
[10] Boles KS, Barchet W, Diacovo T, Cella M, Colonna M. The tumor suppressor TSLC1/NECL-2 triggers NK-cell and CD8+ T-cell responses through the cell-surface receptor CRTAM. Blood. 2005 Aug 1;106(3):779-86.
[11] Chuang YT, Fang LW, Lin-Feng MH, Chen RH, Lai MZ. The tumor suppressor death-associated protein kinase targets to TCR-stimulated NF-κB activation. The Journal of Immunology. 2008 Mar 1;180(5):3238-49.
[12] Intlekofer AM, Takemoto N, Wherry EJ, Longworth SA, Northrup JT, Palanivel VR, Mullen AC, Gasink CR, Kaech SM, Miller JD, Gapin L. Effector and memory CD8+ T cell fate coupled by T-bet and eomesodermin. Nature immunology. 2005 Dec;6(12):1236.
[13] Pipkin ME, Sacks JA, Cruz-Guilloty F, Lichtenheld MG, Bevan MJ, Rao A. Interleukin-2 and inflammation induce distinct transcriptional programs that promote the differentiation of effector cytolytic T cells. Immunity. 2010 Jan 29;32(1):79-90.
[14] Chuang YT, Fang LW, Lin-Feng MH, Chen RH, Lai MZ. The tumor suppressor death-associated protein kinase targets to TCR-stimulated NF-κB activation. The Journal of Immunology. 2008 Mar 1;180(5):3238-49.
[15] Chiu YL, Shan L, Huang H, Haupt C, Bessell C, Canaday DH, Zhang H, Ho YC, Powell JD, Oelke M, Margolick JB. Sprouty-2 regulates HIV-specific T cell polyfunctionality. The Journal of clinical investigation. 2014 Jan 2;124(1):198-208.

Table S7: **FR-AgENCODE transcript positional classification.**

Species	Total #	known		extension		alternative		novel	
		#	% of total	#	% of total	#	% of total	#	% of total
Cattle	84,971	11,736	**13.8**	2,500	2.9	40,813	**48.0**	29,922	**35.2**
Goat	78,091	29,520	**37.8**	2,583	3.3	28,891	37.0	17,097	21.9
Chicken	57,817	15,896	27.5	3,018	5.2	28,065	**48.5**	10,838	18.7
Pig	77,540	23,921	30.8	2,702	3.5	35,062	**45.2**	15,855	20.4

Species	known.mRNA		known.lncRNA		known.otherRNA	
	#	% of known	#	% of known	#	% of known
Cattle	11,576	**98.6**	13	0.1	147	1.3
Goat	26,973	**91.4**	1,163	3.9	1,384	**4.7**
Chicken	14,765	**92.9**	882	**5.5**	249	1.6
Pig	23,701	**99.1**	96	0.4	124	0.5

Species	extension.mRNA		extension.lncRNA		extension.otherRNA	
	#	% of extension	#	% of extension	#	% of extension
Cattle	2,497	**99.9**	0	0.0	3	0.1
Goat	2,351	**91.0**	100	3.9	132	**5.1**
Chicken	2,982	**98.8**	33	1.1	3	0.1
Pig	2,694	**99.7**	5	0.2	3	0.1

Species	alternative.mRNA		alternative.lncRNA		alternative.otherRNA	
	#	% of alternative	#	% of alternative	#	% of alternative
Cattle	40,770	**99.9**	0	0.0	43	0.1
Goat	26,554	**91.9**	984	3.4	1,353	**4.7**
Chicken	27,470	**97.9**	399	1.4	196	0.7
Pig	34,822	**99.3**	226	0.6	14	0.0

Species	novel.mRNA		novel.lncRNA		novel.otherRNA	
	#	% of novel	#	% of novel	#	% of novel
Cattle	4,958	16.6	22,711	75.9	2,253	**7.5**
Goat	2,949	**17.2**	11,617	67.9	2,531	**14.8**
Chicken	2,350	21.7	6,797	62.7	1,691	**15.6**
Pig	2,504	15.8	12,284	77.5	1,067	**6.7**

Table S8: **FR-AgENCODE novel coding genes and their orthology with human**

Species	# FR-AgENCODE genes	# novel FR-AgENCODE genes	# novel coding FR-AgENCODE genes	that can be projected to a human gene	
				#	%
Cattle	34,296	19,324	15,611	166	12.3
Goat	28,537	11,545	16,437	85	6.4
Chicken	20,408	6,597	13,149	78	15.5
Pig	24,570	10,238	14,054	38	9.5

Table S9: **Differentially Expressed (DE) FR-AgENCODE genes.** Number of differentially expressed FR-AgENCODE genes obtained by the two statistical models (see main text and Methods).

Differential analysis model	Tissue 1	Tissue 2	log(Tissue2 / Tissue1)	Number of DE FR-AgENCODE genes			
				Cattle	Goat	Chicken	Pig
1 (tissue pairs)	cd4	cd8	>0	1 450	1 614	1 940	1 211
	cd4	cd8	<0	923	1 056	974	792
	cd4	liver	>0	12 231	11 782	7 426	9 709
	cd4	liver	<0	10 311	7 152	5 134	6 386
	cd8	liver	>0	11 810	11 542	5 899	9 880
	cd8	liver	<0	10 573	7 408	4 331	6 782
2 (tcell vs liver)	cd	liver	>0	12 218	11 773	6 246	10 040
	cd	liver	<0	10 818	7 448	3 929	6 910

Table S10: **lncRNA classification.** Number of classified expressed lncRNAs per species. This table includes monoexonic lncRNAs that represent 57-68% of these transcripts. Loci are bracketed. Fields with an asterisk (*) indicate the existence of lncRNAs unclassified by FEELnc because they are on unassembled contigs; they represent 217, 717, 2,718 and 83 lncRNAs in cattle, goat, chicken and pig respectively and are not listed here.

		Intergenic lncRNAs (loci) – 88-94%					Genic lncRNAs (loci) – 6-12%		
	Total (transcript)	Same strand upstr.	Same strand downstr.	Divergent ≤1kb	Divergent >1kb	Convergent	Exonic antisense	Intronic antisense	Intronic sense
Cattle	22724	5522 (4671)	6438 (5460)	1450 (1070)	4494 (3588)	3328 (2617)	1235 (851)	241 (173)	16 (16)
Goat	13864	2431 (1940)	4050 (3312)	1744 (1241)	2908 (2233)	1966 (1496)	437 (220)	219 (124)	109 (89)
Chicken	7502*	1618 (1390)	2045 (1728)	687 (539)	1194 (983)	1093 (903)	646 (510)	157 (124)	62 (56)
Pig	12587*	2508 (2100)	3539 (3075)	1117 (810)	2466 (2060)	1746 (1436)	935 (669)	216 (178)	60 (58)

Table S11: **FR-AgENCODE transcript coding classification.**

Species	Total #	mRNA		lncRNA		otherRNA	
		#	% ot total	#	% ot total	#	% ot total
Cattle	84,971	59,801	**70.4**	22,724	**26.7**	2,446	2.9
Goat	78,091	58,827	**75.3**	13,864	17.8	5,400	6.9
Chicken	57,817	47,567	**82.3**	8,111	14.0	2,139	3.7
Pig	77,540	63,721	**82.2**	12,611	16.3	1,208	1.6

Species	mRNA.known		mRNA.extension		mRNA.alternative		mRNA.novel	
	#	% of mRNA	#	% of mRNA	#	% of mRNA	#	% of mRNA
Cattle	11,576	19.4	2,497	4.2	40,770	**68.2**	4,958	8.3
Goat	26,973	**45.9**	2,351	4.0	26,554	**45.1**	2,949	5.0
Chicken	14,765	31.0	2,982	6.3	27,470	**57.8**	2,350	4.9
Pig	23,701	37.2	2,694	4.2	34,822	**54.6**	2,504	3.9

Species	lncRNA.known		lncRNA.extension		lncRNA.alternative		lncRNA.novel	
	#	% of lncRNA	#	% of lncRNA	#	% of lncRNA	#	% of lncRNA
Cattle	13	0.1	0	0.0	0	0.0	22,711	**99.9**
Goat	1,163	**8.4**	100	0.7	984	**7.1**	11,617	**83.8**
Chicken	882	**10.9**	33	0.4	399	**4.9**	6,797	**83.8**
Pig	96	0.8	5	0.0	226	1.8	12,284	**97.4**

Species	otherRNA.known		otherRNA.extension		otherRNA.alternative		otherRNA.novel	
	#	% of otherRNA	#	% of otherRNA	#	% of otherRNA	#	% of otherRNA
Cattle	147	6.0	3	0.1	43	1.8	2,253	**92.1**
Goat	1,384	**25.6**	132	2.4	1,353	**25.1**	2,531	46.9
Chicken	249	11.6	3	0.1	196	9.2	1,691	**79.1**
Pig	124	10.3	3	0.2	14	1.2	1,067	**88.3**

Table S12: **Number of ATAC-seq peaks per species.**

Species	Tissue	# ATAC-seq peaks
Cattle	cd4	69,661
	cd8	75,295
	merged	104,985
Goat	cd4	39,526
	cd8	57,084
	liver	14,137
	merged	74,805
Chicken	cd4	38,594
	cd8	49,962
	liver	75,305
	merged	119,894
Pig	cd4	80,745
	cd8	111,457
	liver	25,885
	merged	149,333

Table S13: **Number of differentially accessible (DA) ATAC-seq peaks per species.**

Tissue 1	Tissue 2	log(Tissue2/Tissue1)	Species	Number of DA ATAC-seq peaks
T cell	Liver	>0	Goat	2,780
		<0		2,042
		>0	Chicken	6,663
		<0		6,991
		>0	Pig	5,467
		<0		3,678

Table S14: **Hi-C read pair mapping statistics.** Number of read pairs of different categories. *Initial*: total number of sequenced read pairs. *Reported*: pairs with both reads mapped on the genome. *Valid*: uniquely mapped pairs with an estimated insert size (sum of the distances from the reads to their next downstream HindIII genomic sites) between 20bp and 1Kb. *Valid.rmdup*: valid read pairs after duplication removal that were used to build the interaction matrices. *Trans*: pairs with reads on different chromosomes.

Species	Animal	initial	reported	valid	valid.rmdup	trans
Goat	goat1	192,807,889	164,130,417	94,760,148	77,324,040	31,728,784
	goat2	184,098,994	142,479,273	46,280,100	36,346,806	12,858,516
	goat3	192,081,174	149,494,649	44,712,829	37,115,258	13,227,700
	goat4	178,929,203	135,758,984	38,435,525	31,232,835	12,691,582
Chicken	chicken1	172,356,821	136,854,885	100,067,668	74,551,182	35,227,545
	chicken2	182,654,001	152,646,931	122,151,951	86,044,085	30,151,392
	chicken3	193,520,830	149,648,213	89,183,496	64,824,211	19,603,078
	chicken4	187,696,586	131,460,531	51,115,059	37,069,637	11,768,987
Pig	pig1	168,050,522	139,712,050	111,642,484	82,782,023	25,956,367
	pig2	157,480,346	129,447,277	95,004,214	70,483,985	19,076,359
	pig3	165,285,596	132,403,825	93,115,687	74,144,797	23,560,745
	pig4	165,529,922	131,590,998	82,504,226	62,212,375	18,705,743

Table S15: **Statistics of Hi-C TADs and A/B compartments.**

Feature	Species	Number	Min size	Mean size	Max size	Genomic coverage (Mb)	Genomic coverage (%)
TADs	Pig	10,982	80,000	184,600	4,520,000	2,027.28	83.25
	Chicken	5,362	80,000	148,100	3,520,000	794.28	79.4
	Goat	8,990	80,000	219,800	6,680,000	1,975.96	85.92
A/B comp.	Pig	698	343,300	3,175,000	44,000,000	2,215.84	90.51
	Chicken	578	95,050	1,596,000	10,000,000	922.348	92.21
	Goat	616	426,000	3,412,000	21,000,000	2,101.87	91.4
